# Deep ecomorphological and genetic divergence in Steller's Jays (*Cyanocitta stelleri*, Aves: Corvidae)

**DOI:** 10.1002/ece3.9517

**Published:** 2022-11-30

**Authors:** Carla Cicero, Nicholas A. Mason, Zheng Oong, Pascal O. Title, Melissa E. Morales, Kevin A. Feldheim, Michelle S. Koo, Rauri C. K. Bowie

**Affiliations:** ^1^ Museum of Vertebrate Zoology University of California Berkeley California USA; ^2^ Museum of Natural Science and Department of Biological Sciences Louisiana State University Baton Rouge Louisiana USA; ^3^ Bell Museum of Natural History and Department of Ecology, Evolution and Behavior University of Minnesota Saint Paul Minnesota USA; ^4^ Environmental Resilience Institute Indiana University Bloomington Indiana USA; ^5^ Department of Ecology & Evolution Stony Brook University Stony Brook New York USA; ^6^ Pritzker Laboratory for Molecular Systematics and Evolution, Negaunee Integrative Research Center Field Museum of Natural History Chicago Illinois USA; ^7^ Department of Integrative Biology University of California Berkeley California USA

**Keywords:** clines, contact zone, ecological niche, ecomorphology, integrative taxonomy, microsatellites

## Abstract

The relationship between ecology and morphology is a cornerstone of evolutionary biology, and quantifying variation across environments can shed light on processes that give rise to biodiversity. Three morphotypes of the Steller's Jay (*Cyanocitta stelleri*) occupy different ecoregions in western North America, which vary in climate and landcover. These morphotypes (Coastal, Interior, Rocky Mountain) differ in size, plumage coloration, and head pattern. We sampled 1080 Steller's Jays from 68 populations (plus 11 outgroups) to address three main questions using data on morphology, plumage, genetics (mtDNA, microsatellites), and ecological niches: (1) How do phenotypic and genetic traits vary within and among populations, morphotypes, and ecoregions? (2) How do population‐level differences in Steller's Jays compare with other sister species pairs of North American birds? (3) What can we infer about the population history of Steller's Jays in relation to past climates, paleoecology, and niche evolution? We found substantial morphological, genetic, and ecological differentiation among morphotypes. The greatest genetic divergence separated Coastal and Interior morphotypes from the Rocky Mountain morphotype, which was associated with warmer, drier, and more open habitats. Microsatellites revealed additional structure between Coastal and Interior groups. The deep mtDNA split between Coastal/Interior and Rocky Mountain lineages of Steller's Jay (ND2 ~ 7.8%) is older than most North American avian sister species and dates to approximately 4.3 mya. Interior and Rocky Mountain morphotypes contact across a narrow zone with steep clines in traits and reduced gene flow. The distribution of the three morphotypes coincides with divergent varieties of ponderosa pine and Douglas fir. Species distribution models support multiple glacial refugia for Steller's Jays. Our integrative dataset combined with extensive geographic sampling provides compelling evidence for recognizing at least two species of Steller's Jay.

## Graphical abstract

Three morphotypes of the Steller's Jay (*Cyanocitta* *stelleri*) occupy different ecoregions in western North America, which vary in climate and landcover. We found substantial morphological, genetic, and ecological differentiation among morphotypes. Our integrative dataset combined with extensive geographic sampling provides compelling evidence for recognizing at least two species of Steller's Jay.

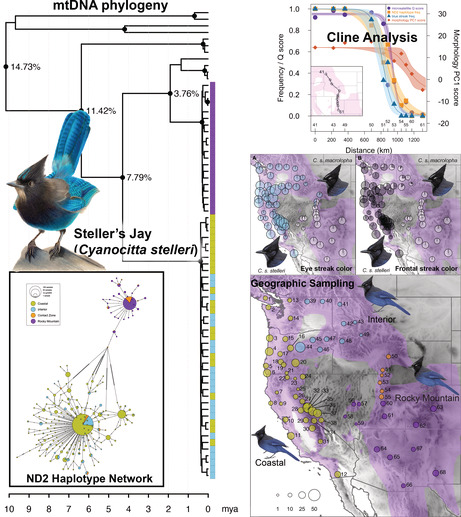

## 1 INTRODUCTION

A cornerstone of ecology and evolution is the interaction between phenotype and environment, which is fundamental to many natural phenomena including molecular evolution, functional morphology, local adaptation and habitat use, intra‐ and interspecific competition, predation and predator avoidance, behavioral ecology, and responses to climate or other anthropogenic changes (Schluter, 2000; Zamudio et al., 2016). For this reason, many organisms show a close association between morphology and ecology, i.e., ecomorphology (Bock, 1994), that reflects divergence and adaptation to different environments. Examples of this association abound across diverse taxa, from insects (Günter et al., 2019; Hughes & Vogler, 2004; Lemic et al., 2016) to fish (Baldasso et al., 2019; Buser et al., 2019; Jacquemin & Pyron, 2016), amphibians (Ficetola et al., 2016; Rebelo & Measey, 2019; Sherratt et al., 2018), reptiles (Kahrl et al., 2018; Kamath & Losos, 2016; Rivera, 2008), mammals (Alvarado‐Serrano et al., 2013; Baier & Hoekstra, 2019; Jones & Law, 2018), and birds (Bravo et al., 2014; Phillips et al., 2020; Pigot et al., 2020; Vanhooydonck et al., 2009). The close connection between morphology and ecology is exemplified at different temporal scales, from rapid evolution in adaptive radiations (e.g., Darwin's finches, Hawaiian honeycreepers; Schluter & Grant, 1984; Tokita et al., 2016) to macroevolutionary change in deep time (Felice et al., 2019; Pigot et al., 2020; but see Phillips et al., 2020). Thus, quantifying geographic variation in phenotypes and genotypes across environments can shed light on the evolutionary processes that give rise to biodiversity.

Morphological traits may exhibit both convergent and divergent patterns that are associated with ecology and behavior, and understanding these associations is important for studying drivers of diversification. For example, bird species living in coniferous forests show convergence in certain morphological traits (body mass, digital pads) that reflect adaptations to conifer needles (Korner‐Nievergelt & Leisler, 2004), and similarities in avian morphological traits predict trophic level, dietary niche, and foraging behavior (Pigot et al., 2020). Conversely, local adaptation to different environments drives ecomorphological divergence and may promote reproductive isolation (Bertrand et al., 2016; Cicero & Koo, 2012; Ribeiro et al., 2014; Shakya et al., 2022). While ecological segregation is thought to drive rapid morphological change in some species (e.g., crossbills, Björklund et al., 2013), it is associated with morphological stasis in others (e.g., *Empidonax* flycatchers, Johnson & Cicero, 2002; *Cinnyris* Double‐collared Sunbirds, McEntee et al., 2016, 2021). Furthermore, different traits may vary in their evolutionary responses to environmental variation, as shown by distantly related clades of *Myrmotherula* antwrens that show similarities in body size but differences in body shape associated with ecology, behavior, and phylogenetic niche conservatism (Bravo et al., 2014). Studies that examine ecological and/or morphological divergence, especially in conjunction with molecular data, are therefore fundamental to elucidating patterns and processes of speciation. This is especially true for cryptic species, which may show subtle morphological differences among populations that occur in different environments (Bickford et al., 2007).

The western United States is well‐suited to studies of ecomorphology because of its geological and paleoclimatic history and associated ecological diversity. Of the 72 ecoregions defined for the contiguous states, 36 (50%) occur from the Pacific Coast to the Rocky Mountains (west of longitude 105°; Olson et al., 2001). These various ecoregions collectively support many diverse species of both plants and animals. In a review of the biodiversity status of each state within the United States of America (Stein, 2002), eight of the 12 western continental states were ranked in the top 20 of all states in terms of species diversity, and 11 were ranked in the top 20 for endemism (Stein, 2002). This diversity is reflected in morphological, ecological, and genetic variation within and among closely related species, with new discoveries of locally adapted populations and divergent lineages uncovered regularly. For example, a new species of crossbill (*Loxia sinesciurus*) restricted to the South Hills and Albion Mountains of southern Idaho was described recently on the basis of morphological, genomic, behavioral, and ecological differences from other members of the Red Crossbill (*L. curvirostra*) complex (Benkman et al., 2009; Parchman et al., 2016). This species has evolved locally to specialize on the cone seeds of Rocky Mountain lodgepole pine (*Pinus contorta latifolia*) and presents a classic case of ecomorphological differentiation driving reproductive isolation from its close relatives. Similar integrative studies have identified ecomorphological divergence and genetic differentiation in other species of western birds, e.g., Dusky Grouse *Dendragapus obscurus* and Sooty Grouse *D. fuliginosus* (Barrowclough et al., 2004); White‐headed Woodpecker *Dryobates albolarvatus* (Alexander & Burns, 2006); California Scrub‐Jay *Aphelocoma californica* and Woodhouse's Scrub‐Jay *A. woodhouseii* (Bardwell et al., 2001; Delaney et al., 2008; Gowen et al., 2014; Peterson, 1993); Canada Jay *Perisoreus canadensis* (Graham et al., 2021; van Els et al., 2012); Oak Titmouse *Baeolophus inornatus* and Juniper Titmouse *B. ridgwayi* (Cicero, 1996, 2004); and Bell's Sparrow *Artemisiospiza belli* and Sagebrush Sparrow *A. nevadensis* (Cicero & Johnson, 2007; Cicero & Koo, 2012).

The Steller's Jay (*Cyanocitta stelleri*) is a common resident bird species of forests and woodlands in western North America and Central America. As many as 16 recognized subspecies are divided into three main groups (Clements et al., 2021; Walker et al., 2020): (1) the Coastal (*stelleri*) Group with four subspecies that range from southwestern Alaska through western British Columbia (including Haida Gwaii) and the United States west of the Cascade Mountains and the Sierra Nevada; some authors (Browning, 2002) also recognize a fifth subspecies *C. s. paralia* in southwestern Oregon and northwestern California, but this taxon is synonymized with *C. s. stelleri* by others (Clements et al., 2021; Walker et al., 2020); (2) the Interior (*diademata*) Group with four subspecies that occur east of the Cascades and Sierra Nevada to the northern and southern Rocky Mountains, extending into northern Mexico; and (3) the Central American (*coronata*) Group with eight subspecies that range from the highlands of northern Mexico to northern Nicaragua. These three groups and associated subspecies are distinguished primarily by plumage coloration (breast and belly, back, head and crest), crest length, and facial patterning (extent and color of streaks on the forehead, which range from blue to white; presence and extent of a white patch or superciliary line above the eye). In addition, subspecies also differ in body size within and among groups, with a trend of larger birds in the north to smaller birds in the south (Walker et al., 2020). Steller's Jays occupy primarily coniferous and mixed coniferous‐deciduous forests but are also found in pine‐oak and oak woodland on exposures with cooler temperatures. Detailed phenotypic and ecological descriptions of Steller's Jay subspecies groups are provided by Walker et al. (2020).

Despite the extensive intraspecific variation, few studies have focused on ecomorphological and/or genetic variation in Steller's Jays. The most complete morphological study to date was conducted by Brown (1963a), who examined 253 specimens (mostly adult males) from across the species' range to investigate ecogeographic factors influencing the crest as a specialized visual signal. Specific attention was paid to body size as measured by wing length, crest length and crest color, frontal (i.e., forehead) streaking, the contrast between the head and body coloration, plumage darkness and saturation, and length of the superciliary line. Results of this study showed strong geographic variation in these traits and associations with both climate and habitat. Brown (1963a) concluded that longer crests and more distinctive facial patterning (white frontal streaks and superciliary line), typical of the Interior Group, are associated with more open, drier forest habitats. Furthermore, he suggested that the frequency of conspecific encounters may be higher in these habitats, resulting in the selection of more conspicuous crests and facial patterns used as visual cues in aggressive encounters. Prior work by Brown (1960) showed that the crest is erected during aggression and serves as an important behavioral signal.

In another study, Bay (2002) compared morphological differentiation in relation to habitat in 156 Steller's Jays representing Rocky Mountain and California populations. In addition to finding significant between‐group variability, results showed a strong association with habitat. For example, similar morphologies associated with feeding and locomotion were found in ecologically similar but geographically distant communities such as ponderosa pine (*Pinus ponderosa*) forest, suggesting an ecomorphological correspondence between sites.

Genetic data on Steller's Jays are currently limited to phylogenetic studies of the family Corvidae (Bonnacorso & Peterson, 2007; Erickson et al., 2005; Saunders & Edwards, 2000) and to a study of eight populations of Steller's Jay in the Pacific Northwest (Burg et al., 2005). The sister species of Steller's Jay is the Blue Jay (*Cyanocitta cristata*), and together they form a lineage that is part of a clade that includes the Pinyon Jay (*Gymnorhinus cyanocephalus*) and scrub‐jays (*Aphelocoma coerulescens*, *A.californica*, *A. woodhouseii*) of North America; also related to this clade are Mexican to South American species of jays in the genera *Calocitta*, *Cyanocorax*, and *Psilorhinus* (Bonnacorso & Peterson, 2007; Erickson et al., 2005; Saunders & Edwards, 2000). Within the Steller's Jay, genetic data based on microsatellites for three northwestern subspecies (*C. s. carolottae*, *C. s. stelleri*, and *S. s. annectens*) found high levels of genetic variation among populations, with the highest divergence between the Haida Gwaii endemic *C. s. carlottae* and mainland subspecies *C. s. stelleri* and *C. s. annectens* (Burg et al., 2005). Although *C. s. stelleri* and *C. s. annectens* were more similar genetically, these taxa also showed population structure with evidence for isolation by distance. The high level of population differentiation found in northwestern Steller's Jays suggests rapid genetic differentiation following postglacial colonization in that region (Burg et al., 2005).

We sampled Steller's Jays extensively across their range in the western United States to investigate broader levels of genetic structure in association with a morphological and ecological variation. Specifically, we focused on populations representing five subspecies that fall into three distinct morphotypes in the Coastal and Interior groups (Table 1). These populations represent the range of ecomorphological variation observed within our focal study area, which extends from the Pacific Coast to the Rocky Mountains. Because our specific goal was to examine phylogeography, population genetic structure, and phenotypic variation within and between these three morphotypes in relation to their environment, we did not sample across the entire range of subspecific and ecogeographic variation in Steller's Jays. Some subspecies and populations not included here have been the subject of prior study (e.g., *C. s. carlottae* and Pacific Northwest populations, Burg et al., 2005), while others are currently part of additional collaborative studies that extend south into Mexico and Central America (Spellman et al., unpublished; McCormack et al., unpublished).

**TABLE 1:** Primary trait characteristics distinguishing three morphotypes of Steller's Jays examined in this study.

Group	Morphotype	Distribution	Subspecies	Crest length	Frontal streaks	Superciliary line
Coastal	Coastal	Pacific slope	*stelleri*, *frontalis*, *carbonacea*	Short	Blue	Absent
Interior	Interior	NW Interior	*annectens*	Short	Blue	Present
Interior	Rocky Mtn.	Rocky Mountains	*macrolopha*	Long	White	Present

*Note*: Group names follow Clements et al. (2021) and Walker et al. (2020).

Steller's Jays in this study occupy 35 of the 36 ecoregions in the western contiguous United States, from the humid forest in the Coast Ranges to Mediterranean climate oak woodlands on the Pacific slope of California, montane coniferous forest from the Cascades‐Sierra Nevada to the Rocky Mountains, and arid pine habitats in the Great Basin; an additional 43 ecoregions intersect the Steller's Jay distribution in Alaska, Canada, and Mexico. Thus, Steller's Jays experience a breadth of bioclimatic and ecological conditions that have contributed to its diversification. Specifically in the western United States, these ecoregions have undergone significant climatic and ecological fluctuations (Gavin, 2009; Shinneman et al., 2016) and experienced paleoclimatic events that have played an important role in divergence, speciation, and trait evolution in temperate birds (Friis et al., 2018; Johnson & Cicero, 2004; Lawson & Weir, 2014; Weir & Schluter, 2004). We examined variation in morphology, plumage, DNA, and environment to quantify patterns of differentiation within and among populations and morphotypes of Steller's Jays to address three main questions: (1) How do phenotypic and genetic traits vary within and among populations, morphotypes, and ecoregions? (2) How do population‐level differences in Steller's Jays compare to other sister species pairs of North American birds? and (3) What can we infer about the population history of Steller's Jays in relation to past climates, paleoecology, and niche evolution? In addition, we evaluate these data in the context of Brown's (1963a) hypothesis that visual signaling traits (e.g., facial markings, crest length) have played an important role in Steller's Jay diversification.

## 2 MATERIALS AND METHODS

We used an integrative approach to address the focal questions in our study. Data integral to understanding the results of our analyses are presented as figures or tables in the main text or in accompanying appendices (Figures A1, A2, A3, A4, A5, A6, A7, A8, A9, A10, A11, A12, A13, A14; Tables A1, A2, A3, A4, A5, A6, A7, A8, A9, A10). Additional supporting data are provided in supplementary materials (Tables [Link], [Link]) and deposited in Dryad or Zenodo, along with the R code used to perform the analyses detailed below.

### 2.1 Sampling

**FIGURE 1:**
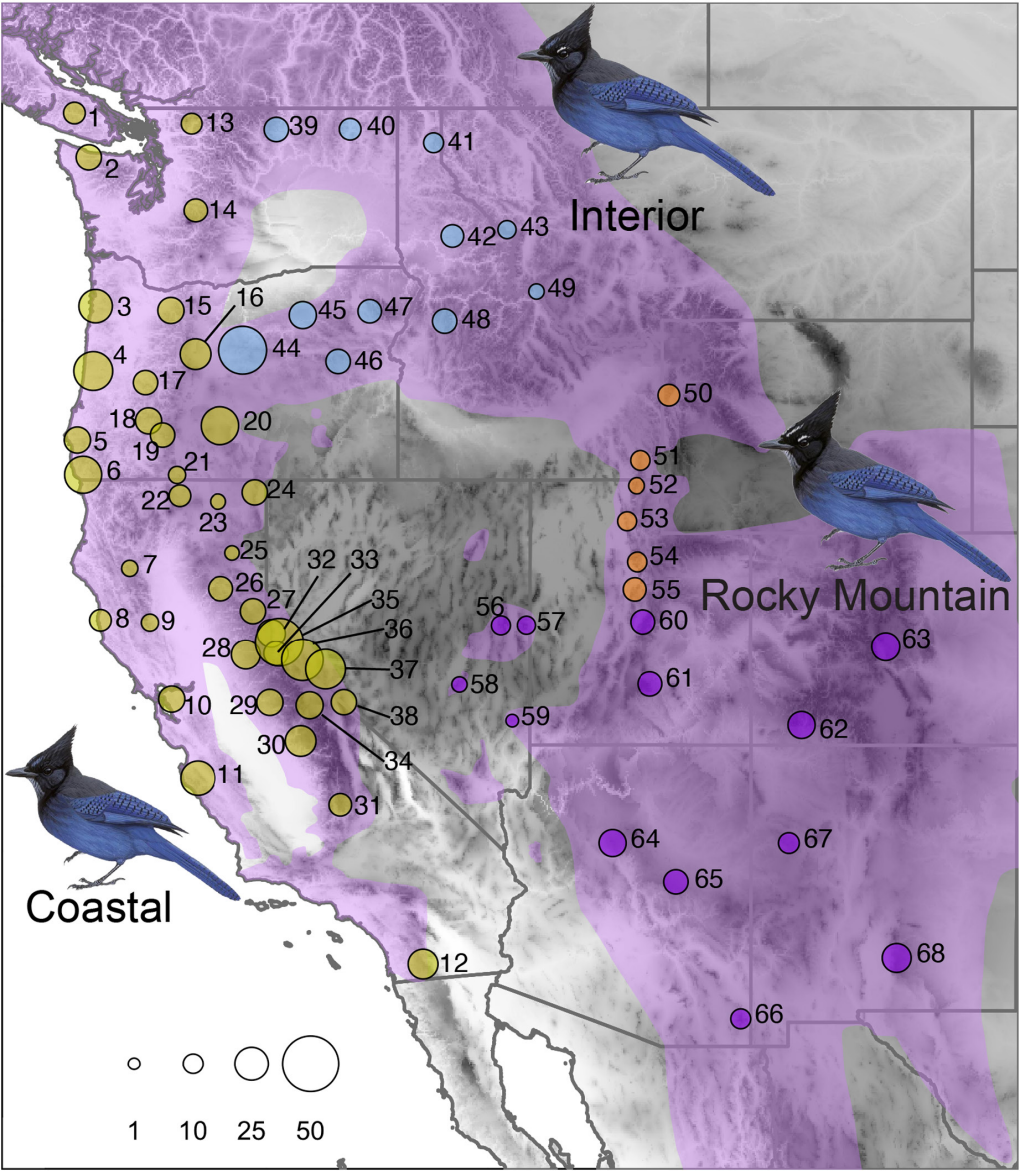
Sampling locations for Steller's Jays included in this study. Purple shading represents their range based on BirdLife International, edited to reflect known occurrences. Numbers correspond to the population numbers listed in Table S1. Circle colors correspond to the three distinct morphotypes (Table 1) present in the study area (yellow—Coastal; blue—Interior; purple—Rocky Mountain). Orange circles highlight a putative contact zone between Interior and Rocky Mountain populations based on the STRUCTURE analysis of microsatellites (Figure 6). The circle sizes correspond roughly to the sample size at each location (see scale on the bottom left). The Steller's Jay plate on the bottom left (*C. s. stelleri*) represents the Coastal morphotype with a short crest, blue frontal streaks, and no supercilium. The Steller's Jay on the top right (*C. s. annectens*) represents the Interior morphotype with a short crest, blue frontal streaks, and white supercilium. The Steller's Jay on the bottom right (*C. s. macrolopha*) represents the Rocky Mountain morphotype with a long crest, white frontal streaks, and a white supercilium. Elevation is shown in grayscale with darker gray representing higher elevation. Illustrations are reproduced from https://birdsoftheworld.org with permission from Lynx Edicions.

We sampled 1080 *C. stelleri* and assigned 1075 of those individuals to 68 populations from across the species' range in the western United States and Vancouver Island, British Columbia (Figure 1); the remaining five individuals were from isolated locations with low sampling. Sampling of these 68 populations focused on five subspecies from the Coastal (*C. s. stelleri*, *C. s. frontalis*, *C. s. carbonacea*) and Interior (*C. s. annectens*, *C. s. macrolopha*) subspecies groups (Clements et al., 2021; Walker et al., 2020). In addition, we included 11 specimens of other *C. stelleri* subspecies or related species as outgroups: *C. s. coronata* from Mexico (1), *C. s. ridgwayi* from Mexico (1) and Guatemala (2), *C. cristata* (Blue Jay, 3), *Aphelocoma californica* (California Scrub‐Jay, 1), *Aphelocoma coerulescens* (Florida Scrub‐Jay, 1), and *Gymnorhinus cyanocephalus* (Pinyon Jay, 2). Of the 1091 total individuals sampled for this project (1080 ingroup samples of *C. stelleri* plus 11 outgroup samples), 1053 specimens are housed in the Museum of Vertebrate Zoology (MVZ), University of California, Berkeley. Data for another 33 specimens were obtained from specimens at the Burke Museum of Natural History and Culture, the University of Washington (*n* = 22, tissues), and the Museum of Southwestern Biology, University of New Mexico (*n* = 11, tissues plus vouchers). We also made use of five sequences sourced from GenBank. Details for the total dataset are given in Table S1.

### 2.2 Morphological data collection and analyses

We used Fowler Ultra‐Cal digital calipers to measure standard morphological characters on 1035 specimens (Table S2) prepared as study skins representing vouchers for the genetic samples; we limited our analyses to skins associated with tissue samples in order to associate the morphological data directly with the molecular data. We only measured birds of known sex that had molted into at least their first‐year (non‐juvenile) plumage. Linear measurements (mm) included wing length, tail length, tarsus length, length of the middle toe, bill length, bill depth, and bill width (see Cicero, 1996:12 for details on methodology). We added the measurements for tarsus plus toe length to form a single character for analysis because the transition from tarsus to toe was not always clear on the specimens. We also measured crest length from the point of insertion of the longest crest feather to its tip. We were able to do this for all but a small (7%) number of birds, which had crests that were either too worn or molting heavily. Finally, we recorded the mass (grams) for every specimen from the data on the original specimen label.

We recorded the sex and age of each bird from the specimen label data. Sex was based on examination of reproductive organs during preservation and was scored as male, female, or unknown/questionable. Age was recorded in two ways: (1) extent of skull ossification noted when the specimen was prepared; and (2) examination of tail shape, which can be used to distinguish first‐year from older birds. Steller's Jays have a partial first prebasic (first year) molt, which involves the replacement of body but not wing and tail feathers (Walker et al., 2020). A small percentage (5%) of birds could not be aged easily by the tail shape, but they could be aged by ossification. Likewise, a few birds (less than 1%) could be aged by the tail but not by the skull. Age data were used to score each specimen as an adult (skull 100% ossified, adult tail) or immature (skull not fully ossified, first‐year tail).

We recorded two plumage characters that are diagnostic of the different Steller's Jay morphotypes: (1) The color of frontal (forehead) streaking. Birds were scored as having either blue or white frontal streaks. Although Pacific Slope and Northwestern Interior birds have mostly blue streaking, close examination of the feathers revealed varying amounts of white mixed with blue. This contrasts with Rocky Mountain birds that have white streaking with no blue. Thus, if the streaks contained any amount of blue, they were scored as being “blue.” (2) The extent of the white superciliary line above the eye. We measured the length and width of the line using digital calipers and then multiplied width by length to estimate the eye line area. Birds with no white line were scored as “0” for both length and width. We recognize that a more quantitative approach to measuring plumage coloration (body plumage, eye‐streak color) and patterning (size of frontal streaks) in Steller's Jays would be beneficial (Mason & Bowie, 2020), but these two characters provided a basic view of variation in traits that are important for distinguishing morphotypes.

To explore morphological variation among Steller's Jays by sex, age, morphotype, and population, we conducted a Principal Components Analysis (PCA) using the function princomp() from base R (R Core Team, 2020) and then implemented linear mixed models (LMMs) using the lme() function from the R package nlme v. 3.1.148 (Pinheiro et al., 2020). We included sex (male, female), age class (first year, adult), and morphotype (Coastal, Interior, Rocky Mountain) as fixed effects in all the models, and population as a random grouping effect because multiple individuals were typically sampled from the same population or locality. Using the R package MuMIn v 1.43.17 (Bartón, 2019), we calculated the marginal and conditional coefficients of determination ($R_{m}^{2}$ and $R_{c}^{2}$, respectively) for each linear mixed model to assess whether including sampling locality as a random effect impacted the fit of the model (Nakagawa & Schielzeth, 2013). For the LMMs, we omitted six populations from a putative contact zone (Figure 1, Table S1) as they showed evidence of admixture from our ND2 haplotype network, STRUCTURE analysis, and phenotypic data (see below). We also generated box plots illustrating interquartile ranges for each morphological character and principal component axis for our fixed effect groupings (sex, age class, morphotype) and for each of the 68 populations. For our fixed effect groupings, we conducted a Tukey's honestly significant difference test with the R package agricolae v1.3–5 (de Mendiburu, 2020) to compare the mean values of different groupings. Finally, we used the R package MASS v7.3‐54 (Venables & Ripley, 2002) to conduct a Discriminant Function Analysis (DFA) with cross‐validation using all continuous characters to quantify diagnosability among the three morphotypes (contact zone excluded).

### 2.3 DNA laboratory methods

We extracted genomic DNA from frozen tissue samples using either a salt extraction protocol (Aljanabi & Martínez, 1997) or the Qiagen DNeasy Kit following the manufacturer's recommendations (Qiagen, Valencia, California). Following extraction, we used PCR to amplify the complete mitochondrial NADH Dehydrogenase subunit II (ND2) gene using primers L5204 and H6312 (Cicero & Johnson, 2001) with the following protocol: 94°C for 3 min, 35 cycles of 94°C denaturation for 30 s, 54°C annealing for 30 s, 72°C elongation for 45 s, and a final 10 min elongation at 72°C. We used 3 μl of each 10 μl PCR product for visualization on a 1% agarose gel stained with ethidium bromide. We then purified the remaining PCR products using 1 μl of 1:10 diluted ExoSAP solution incubated at 37°C for 30 min, followed by a denaturation step at 80°C for 15 min. We cycle‐sequenced the purified products using Big Dye terminator chemistry (Applied Biosystems, Foster City, California), purified the cycle‐sequencing products using Sephadex columns, and ran those on an ABI 3730 DNA Analyzer (Applied Biosystems). We assembled, checked, edited, and aligned all sequences using CodonCode Aligner version 4.2.7 (CodonCode Corporation).

We used 12 polymorphic microsatellite markers developed specifically for *C. stelleri* (Bowie et al., 2022) to genotype 1075 of the 1080 ingroup samples that we also sequenced for ND2 (Table S4). Of the five excluded individuals, four were analyzed for ND2 only (from GenBank sequences) and one (MVZ:Bird:179153) was dropped due to suspected contamination. All 12 loci were tetranucleotide repeats. We amplified the marker regions by PCR using the following reagents: 1× PCR Buffer (20 mM Tris HCl, 50 mM KCl, pH 8.4), 1.5 mM MgCl_2_, 10 mM of each dNTP, 10 mg/ml of BSA, 0.6 U of Taq polymerase, and approximately 10–30 ng genomic DNA. The thermocycling profile of the PCR reactions was 94°C for 2 min followed by 30–35 cycles of 94°C denaturation for 15 s, a locus‐specific annealing temperature (53–57°C) for 15 s, and 72°C elongation for 15 s. We isolated 2 μl of the 10 μl PCR products for visualization on 1% agarose gels stained with ethidium bromide. We then mixed the PCR products with a GS‐500 LIZ size standard (Applied Biosystems) and formamide, denatured the mixture at 94°C for 2 min, and loaded the mixture on an ABI 3730 DNA Analyzer. For 5 of the 12 markers, we cleaned the PCR product with T4 DNA polymerase (0.21 U) prior to loading to remove peaks of 3′ A nucleotide additions. We scored the resulting alleles for all 12 loci and 1075 individuals using GeneMapper 4.0 (Applied Biosystems).

### 2.4 Phylogenetic analysis

We generated phylogenies from the ND2 alignment using both Bayesian and maximum likelihood methods for a subset of the Steller's Jays sampled for this study plus the 11 additional outgroup samples. Because of the large number of Steller's Jays in the entire dataset, we randomly selected two individuals from 10 populations representing each of the three morphotypes (total of *n* = 60 individuals). We determined the best‐fitting model of nucleotide substitution with the programs PartitionFinder v2.1.1 (Lanfear et al., 2016) and PhyML (Guindon et al., 2010), and used the Bayesian information criterion to select from six models with each of the three codon positions as either linked or unlinked: JC, JC + I, HKY, HKY + I + G, GTR, GTR + G. The best performing model was HKY + I + G with separate, unlinked models inferred for each codon position partition. Using BEAST v2.4.5 (Bouckaert et al., 2014; Drummond & Rambaut, 2007), we then inferred a Bayesian phylogeny with a strict clock model of 2.1% divergence per million years (Weir & Schluter, 2008) that was linked across codon positions (see xml input file on Dryad for complete input settings). We ran three separate Bayesian BEAST analyses for 1 × 10^7^ generations, from which we discarded the first 10% of generations as burn‐in. We then combined the post‐burn‐in output of each run and generated a maximum clade credibility tree. We also used RAxML v7.4.2 (Stamatakis, 2006) to infer a maximum‐likelihood phylogeny. For RAxML, we used a GTR + G model of substitution, performed a rapid bootstrap analysis, and searched for the best‐scoring maximum‐likelihood tree within one program run (‘‐f a’ option). Finally, we used the R package ape v5.0 (Paradis & Schliep, 2019) to calculate the uncorrected genetic distance among clades.

### 2.5 Population genetic analyses

We generated a minimum spanning network of the ND2 haplotypes with PopART version 1.7 (Bandelt et al., 1999; Leigh & Bryant, 2015; see Table S3 for the Nexus input file). To reflect the geographic distribution of samples, we labeled each haplotype by morphotype as listed in Table S1.

**FIGURE A1:**
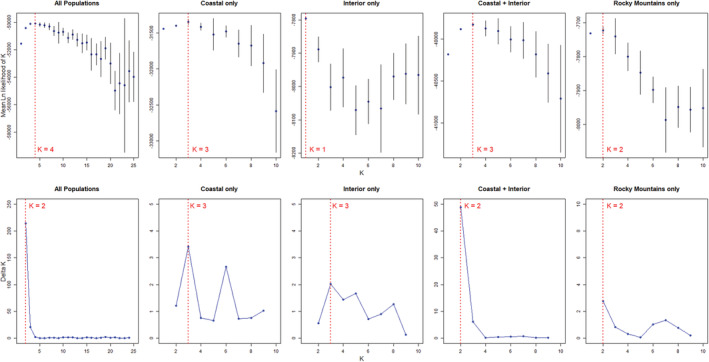
Plots of likelihood and Delta *K* values for different numbers of clusters (*K*) based on 12 microsatellite loci. Separate analyses were run for all populations combined and for different combinations based on morphotype (Coastal only, Interior only, Coastal plus Interior, Rocky Mountain only). Vertical lines in red indicate the optimal *K* value for that analysis.

We used a Bayesian approach to determine the level of population structure in the microsatellite data using the program STRUCTURE v2.3.4 (Hubisz et al., 2009; Pritchard et al., 2000). To estimate the number of genetically distinct clusters (*K*), we used an admixture model with correlated allele frequencies and performed 10 independent runs of 5 × 10^5^ MCMC iterations after a burn‐in period of 1 × 10^5^ iterations, using sampling locations as priors (LOCPRIOR). We assessed the likely number of *K* based on the inspection of plots (Figure A1) for mean log likelihood of *K* (Ln *P*(*K*); Pritchard et al., 2000) and Delta *K* (Δ*K*; Evanno et al., 2005), both of which were obtained through Structure Harvester (Earl & vonHoldt, 2012). For the relevant runs, we used CLUMPP v1.1.2 (Jakobsson & Rosenberg, 2007) to generate mean membership coefficients for each individual sample using the “FullSearch” method (option 1).

To further visualize population clustering, we used the R package ADEGENET v2.1.1 (Jombart, 2008) to perform a discriminant analysis of principal components (DAPC, Jombart et al., 2010) on the microsatellite individual‐genotype matrix. DAPC is a computationally fast, multivariate method for identifying the number of genetic clusters within a large dataset by maximizing between‐group variation while minimizing within‐group variation (Jombart et al., 2010). To minimize overfitting, we performed an initial DAPC and evaluated the optimal number of principal components that would maximize the a‐score; we used this number to select the number of principal components to retain in a subsequent re‐analysis of the same dataset (Jombart & Collins, 2015). We then constructed a scatterplot of individuals by morphotype along the first two discriminant function axes to visualize separation based on microsatellites. We excluded populations from the putative contact zone, resulting in *n* = 1013.

We used ARLEQUIN v3.5.2.2 (Excoffier & Lischer, 2010) and DnaSP v6.12.03 (Rozas et al., 2017) to estimate the following population genetic statistics for the ND2 dataset, with samples grouped by morphotype (Table 1; Table S1): (1) nucleotide diversity and its variance (Nei, 1987); (2) genetic diversity estimators *θ* by segregating sites (*S*) and number of pairwise differences (*π*); and (3) Fu's *F*_S_ (Fu, 1997) and Tajima's *D* (Tajima, 1989) statistics, which test for selective neutrality. We included invariable sites and sites with alignment gaps in our dataset. Again, we excluded populations from the putative contact zone, resulting in *n* = 1019.

We also used ARLEQUIN to estimate observed and expected heterozygosity (*H*_O_ and *H*_E_ respectively) and tested for departure from Hardy–Weinberg equilibrium at each of the 12 microsatellite loci (Excoffier & Lischer, 2010). We grouped samples by morphotype and excluded the contact zone populations. Results from the Hardy–Weinberg equilibrium test were corrected for multiple comparisons using the Holm–Bonferroni sequential correction (Holm, 1979). We tested all populations and loci for linkage disequilibrium using GENEPOP v4.2 (Raymond & Rousset, 1995; Rousset, 2008). To account for the effects of population sampling on the number of alleles detected, we calculated the allelic richness (RS) for each locus in our populations using FSTAT version 2.9.3.2 (Goudet, 2002). We also calculated the number of private microsatellite alleles across all populations using the “Private Allele List” option in GenAlEx 6.5 (Peakall & Smouse, 2006, 2012).

Finally, we used the 12 microsatellite loci to visualize genetic connectivity and variation among our sampled populations with the program EEMS (Estimated Effective Migration Surfaces, Petkova et al., 2016). We assigned individuals to one of 500 demes (i.e., equally spaced vertices) within the geographic extent of our study, and subsequently ran three separate Bayesian chains for 2 × 10^6^ generations, of which we discarded the first 25% as burn‐in. We examined stationarity and congruence among each of our runs and then visualized the output with the package rEEMSplots v0.9 (Petkova et al., 2016).

### 2.6 Cline analysis

We used the R package HZAR v0.2.5 (Derryberry et al., 2014) to fit geographic clines for mtDNA, microsatellite, and morphological data across 11 populations from northern Idaho to southern Utah (41, 43, 49, 50–55, 60–61; Figure 1, Table A1) that showed divergence and evidence of admixture across the putative contact zone. This allowed us to estimate the center and width of clines using an MCMC algorithm to visualize changes in molecular and morphological variation and to determine potential mismatches between datasets. We calculated linear distances (rounded to the nearest 0.1 km) between the 11 populations using decimal coordinates and compressed all distance measurements into a single line on HZAR.

We fitted clines to PC1 scores of morphology, frequency of blue streaking, mtDNA haplotype frequencies, and Q scores obtained from STRUCTURE analyses of the microsatellite genotypes (see McEntee et al., 2016 and citations therein for use of Q scores in cline analyses). We fitted a total of 5 possible cline architectures that included a sigmoid curve with: (1) no exponential tails; (2) a right‐flanking exponential tail; (3) a left‐flanking exponential tail; (4) mirrored exponential tails; and (5) independent exponential tails on both sides. For each tail shape, we allowed the PMIN and PMAX (or “scaling”) to be excluded (PMIN = 0 and PMAX = 1), fixed to the observed minimum and maximum, or free to vary. Thus, we tested a total of 15 possible models.

For each model, we ran a chain of 1 × 10^6^ generations and discarded the first 5 × 10^5^ generations as burn‐in to optimize the covariance matrix used for an additional three independent MCMC chains (Derryberry et al., 2014). We ran the three subsequent chains for each model for a total of 9 × 10^6^ generations and assessed convergence by inspecting the MCMC traces (Derryberry et al., 2014). We selected the best model using the corrected Akaike Information Criterion (AIC_c_).

### 2.7 Comparison of ND2 divergence to sister species pairs

To place our study in a broader context, we extracted the raw percent divergence in ND2 sequences for a set of 30 North American avian species pairs (Table A2) and compared those values with the ND2 divergence between Coastal/Interior and Rocky Mountain Steller's Jays. We combined Coastal and Interior morphotypes for this analysis because together they form a separate mtDNA group from the Rocky Mountain populations (see below). We used the R package rentrez v1.2.3 (Winter, 2017) to query the National Center for Biotechnology Information (NCBI) nucleotide database and downloaded all available ND2 sequences for each species pair. After aligning each set of ND2 sequences using MUSCLE (Edgar, 2004) as called from the R package ape v5.4.1 (Paradis & Schliep, 2019), we calculated the minimum, mean, and maximum raw pairwise sequence divergence for each species pairwise comparison. We excluded sequences from individuals known to be in a contact zone between species (e.g., *Aphelocoma californica* and *A. wollweberi*, Gowen et al., 2014).

### 2.8 Environmental variation

#### 2.8.1 Occurrence records

To examine how Steller's Jays are distributed across environmental space, we downloaded 1,032,960 specimen‐based and observational occurrence records from 118 datasets accessed through the Global Biodiversity Information Facility (GBIF.org, 26 April 2019). Using the R package sf (Pebesma, 2018), we subsequently excluded points that fell outside of the species' known geographic range, buffered by one degree, as well as those with a coordinate uncertainty (if provided) greater than 5 km. This resulted in 1,024,155 occurrence records.

#### 2.8.2 Sampling bias

Spatial sampling bias, which is pervasive in museum collections and observational databases (Beck et al., 2014), may occur when the relative sampling of environmental space is biased by the data collection protocol and does not represent the true environmental preferences of a species. Such bias is known to reduce the accuracy of species distribution models, especially when there is a lack of true absences (Boria et al., 2014; Fourcade et al., 2014; Kramer‐Schadt et al., 2013; Phillips et al., 2009; Syfert et al., 2013). We took two approaches to lessen the impact of spatial sampling bias. First, we applied a proximity filter using the geoThin function in the R package enmSdm v0.5.3 (Smith, 2020) such that occurrences were no closer than 10 km from each other. When possible, we prioritized specimen‐based occurrences over observational records. These filters resulted in 8262 occurrence records (Table S5). Second, we evaluated several anthropogenic predictors that could bias sampling, including distances to roads, populated places, urban areas, and protected areas. If such factors contribute to sampling bias in occurrence records, we wanted to account for the same bias in pseudo‐absences by introducing it to the sampling of environmental space (Merow et al., 2016). We downloaded these datasets as vector data from www.naturalearthdata.com and found that species occurrences had the lowest mean distance to roads. Thus, we selected this anthropogenic predictor to represent sampling bias. After converting this distance to roads vector dataset to a grid format, we coded all grid cell values on a scale of 0 (minimal distance to roads) to 1 (maximal distance to roads). We then sampled 100,000 points in proportion to these grid cell values, such that coordinates near roads were more likely to be sampled. This set of points was used as pseudo‐absences in subsequent species distribution modeling.

#### 2.8.3 Environmental predictors

We acquired climate data in the form of monthly precipitation and temperature grids from the CHELSA dataset v1.2 (Karger et al., 2017, 2018). In addition to current climate, data were downloaded for the last glacial maximum (LGM, 21,000 years ago) under the PMIP3 NCAR‐CCSM4 global circulation model, and for the period 2040–2060 under the CMIP5 NCAR‐CCSM4 global circulation model (RCP4.5 scenario). All climate data were downloaded at a resolution of 30 arc‐seconds and resampled to 60 arc‐seconds (~ 2 km per grid cell).

For each time period, we generated 19 bioclimatic variables from the monthly CHELSA climate grids with the *biovars* function in the R package dismo v1.1–4 (Hijmans et al., 2017) and generated a set of 14 additional bioclimatic variables with the R package envirem v2.1 (Title & Bemmels, 2018). We used the R package raster v3.4–5 for general climate data manipulation (Hijmans, 2020). We also incorporated consensus landcover variables for the current climate period (landcover is not available for the LGM or future) in the form of nine landcover types (Tuanmu & Jetz, 2014); we excluded three landcover types (managed vegetation, urban, and open water) that were less relevant for our study. The full list of variables has been compiled in Table A3.

#### 2.8.4 Species distribution modeling

We first used the vifcor() function in the R package usdm v1.1‐18 (Naimi et al., 2014) to reduce the collinearity in our set of predictor variables by assessing pairs of predictors that had a correlation coefficient greater than 0.8, and dropped the predictor with a greater variance inflation factor in each case. We then fit species distribution models with the maxnet algorithm (Phillips et al., 2017), which is an implementation of the popular MaxEnt program as an infinitely‐weighted logistic regression.

Overly parameter‐rich models run the risk of being overfit to the data and of being less transferable to other time periods (Merow et al., 2014; Radosavljevic & Anderson, 2014; Warren & Seifert, 2011; Wright et al., 2014). Therefore, we further reduced the number of predictor variables and selected the optimal set of feature classes and smoothing parameters via a backward selection procedure that utilizes the corrected Akaike Information Criteria (AICc) as the evaluation criterion (Merow et al., 2013; Warren et al., 2014; Warren & Seifert, 2011). We started with all predictor variables (post initial reduction), identified the feature classes and smoothing parameters that led to the best AICc, and dropped the predictor variable that contributed the least to the model according to MaxEnt permutation importance. We repeated this until all included predictors had a permutation importance of at least 1%. For feature classes, we considered all combinations of linear, quadratic, and hinge features. We excluded product and threshold features because those were not found to be particularly important in the more recent versions of MaxEnt (Phillips et al., 2017). We tested smoothing parameters from 1 to 12 with increasing increments of 0.5. This led to 161 parameter combinations. We ran these procedures for occurrences associated with each morphotype using a combination of existing and custom functions in R, with the help of packages enmSdm (Smith, 2020), dismo (Hijmans et al., 2017), maxnet (Phillips et al., 2017), and raster (Hijmans, 2020).

#### 2.8.5 Discriminant function analysis

We performed a discriminant function analysis (DFA) with the environmental dataset using the MASS package v7.3‐51 in R (Venables & Ripley, 2002) to determine the ability of climate and landcover variables to classify Steller's Jay occurrences into their respective morphotypes. We used the same reduced set of climate predictors selected for the SDM analyses as input variables and combined all forest‐related classes (landcover classes 1–4) by summing the percent landcover to represent a single variable for closed habitat. We further used the DFA predictor loadings to identify the most influential variables in separating Steller's Jay morphotypes.

## 3 RESULTS

### 3.1 Morphological variation

The PCA of morphological variation (Table A4) showed that the first axis loaded positively with body size (wing length, tail length, mass) and accounted for 43.42% of total variation. The second PCA axis, which accounted for 23.98% of the total variation, loaded positively with wing length, tail length, and crest length, and negatively with mass. Bill dimensions (length, width, or depth) did not load highly with either of the first two principal component axes.

**FIGURE 2:**
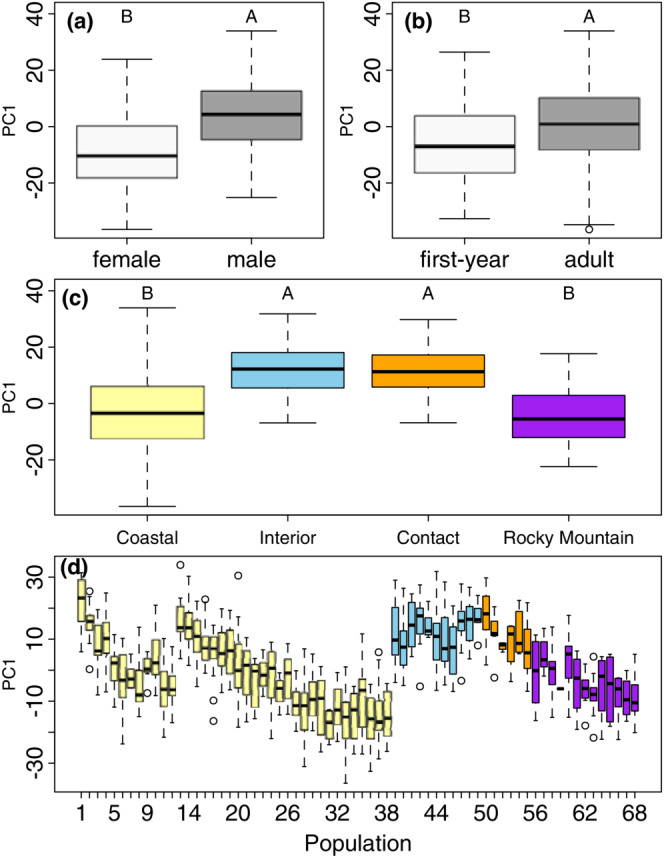
Box plots of the first principal component scores among (a) sexes, (b) age classes, (c) morphotypes, and (d) populations. Box plots show quartile ranges and circles indicate outliers, which fall outside 1.5 times the interquartile range above the upper quartile or below the lower quartile. Letters above each boxplot indicate post hoc groupings following multiple comparisons of means via Tukey's honestly significant difference test with an alpha value of .05.

Results from the linear mixed model analysis of morphological variation using principal component vectors (Table 2, Figure 2) showed statistically significant differences (*p* < .001) between sex and age groups, with males being larger than females (Figure 2a) and adults being larger than first‐year birds (Figure 2b). In our pairwise comparisons of morphotypes across all populations within the four groupings (Figure 2c), Interior and Contact Zone birds were significantly larger (i.e., higher PC1 scores) than Coastal and Rocky Mountain birds (*p* < .05, Table 2, Figure 2c), whereas Coastal and Rocky Mountain birds did not differ significantly in size from each other (*p* > .05, Table 2, Figure 2c), nor did Interior and Contact Zone birds (Figure 2c, Table 2).

**TABLE 2:** Results from the linear mixed model of variation in the first principal component axis of morphological variation in Steller's Jays, which loads positively with overall body size.

Effect	Value	*T* value	*p*‐Value	$R_{m}^{2}$	$R_{c}^{2}$
(Intercept)	−12.74 ± 1.5	−8.47	<.001	.40	.78
Sex (Male)	11.91 ± 0.45	26.32	<.001		
Age (Adult)	3.15 ± 0.76	4.15	<.001		
Morphotype (Interior)	14.55 ± 2.76	5.27	<.001		
Morphotype (Rocky Mtn.)	−1.6 ± 2.63	−0.61	.55		

*Note*: Population was included as a random grouping effect because multiple individuals were sampled from the same population or locality. *p*‐Values below the .05 threshold are considered statistically significant. The effects of each factor are compared with a base model that describes variation among female, immature birds from coastal morphotype populations. The marginal ($R_{m}^{2}$) and conditional ($R_{c}^{2}$) coefficients of the linear mixed model are also shown.

**FIGURE A2:**
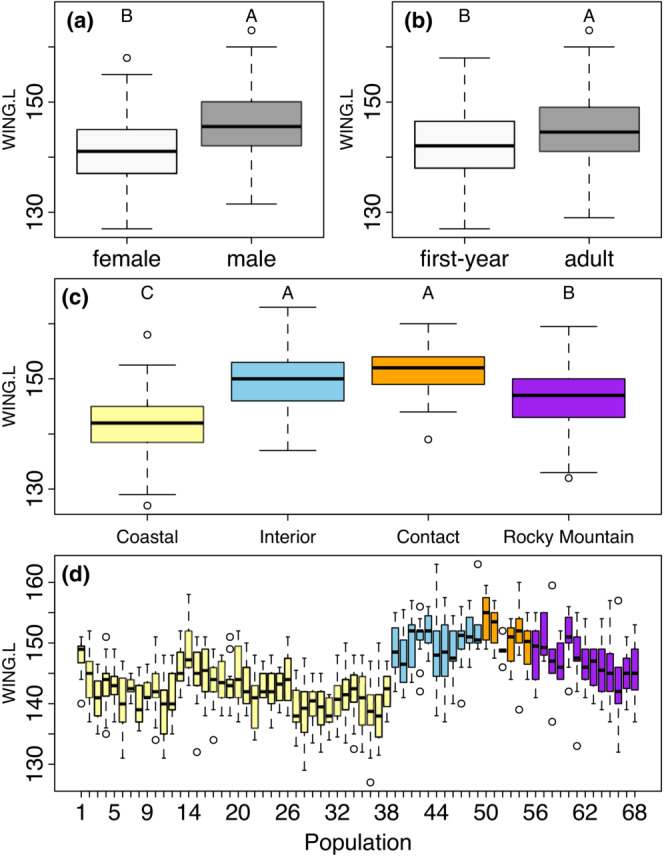
Box plots of wing length measurements (mm) for comparisons by sex (a), age (b), morphotype (c), and population (d).

**FIGURE A3:**
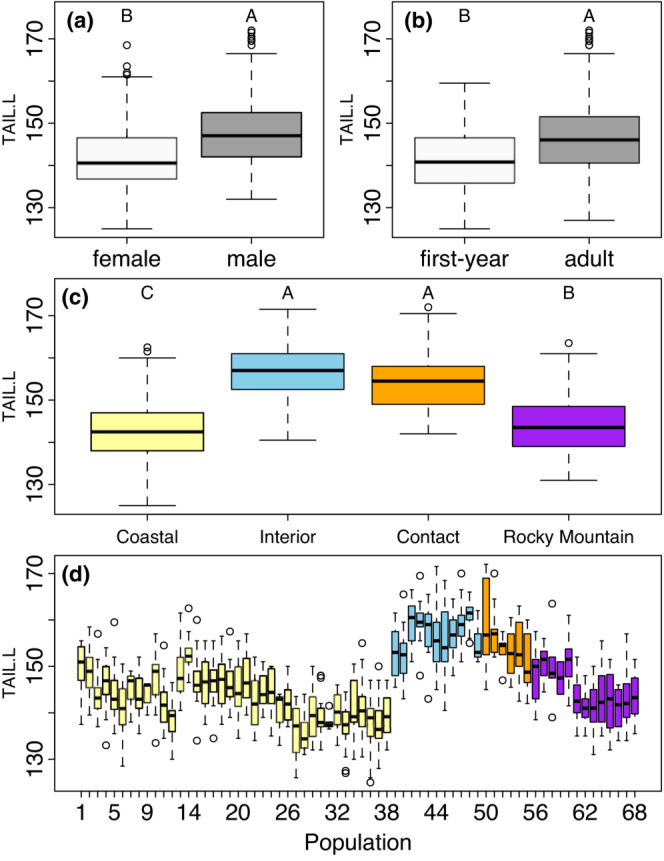
Box plots of tail length measurements (mm) for comparisons by sex (a), age (b), morphotype (c), and population (d).

**FIGURE A4:**
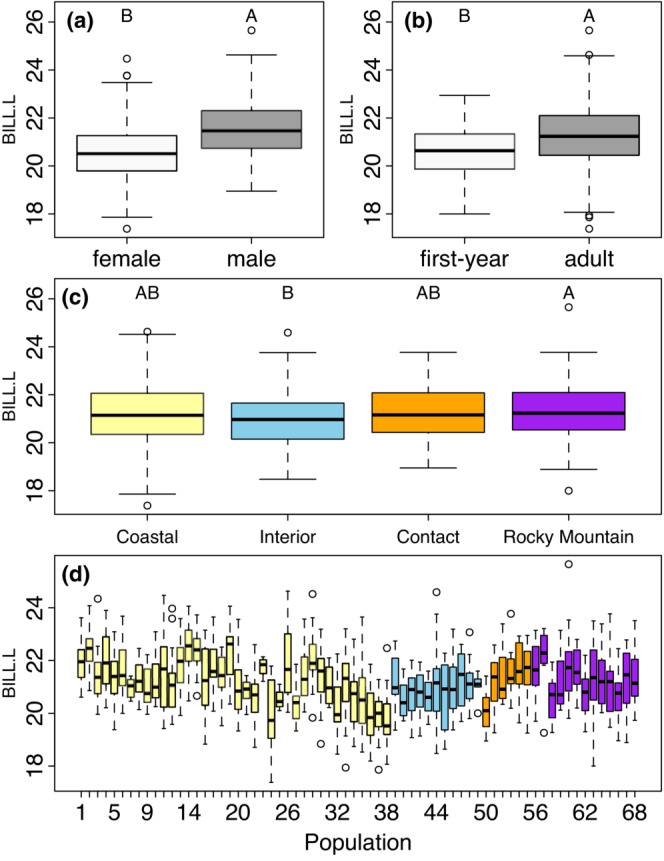
Box plots of bill length measurements (mm) for comparisons by sex (a), age (b), morphotype (c), and population (d).

**FIGURE A5:**
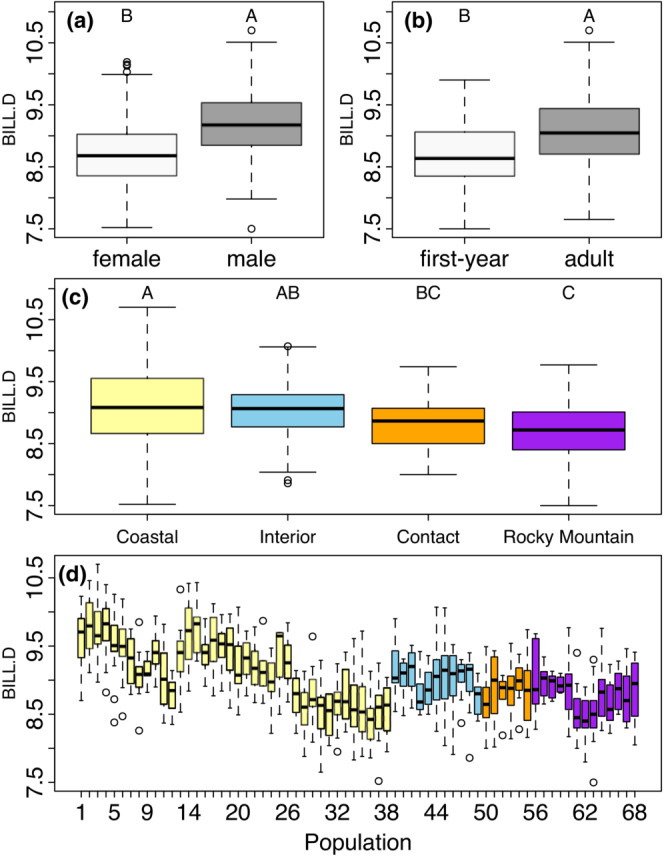
Box plots of bill depth measurements (mm) for comparisons by sex (a), age (b), morphotype (c), and population (d).

**FIGURE A6:**
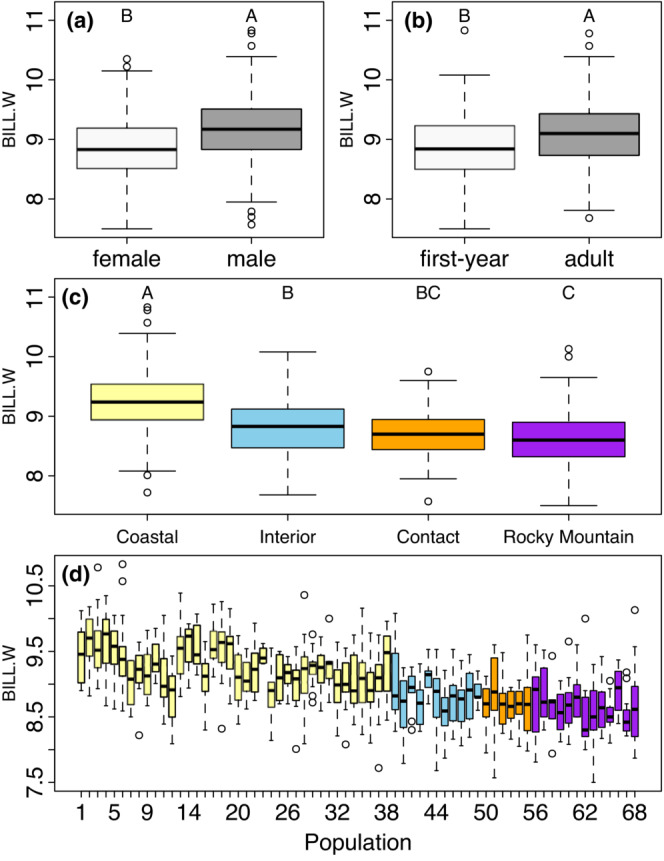
Box plots of bill width measurements (mm) for comparisons by sex (a), age (b), morphotype (c), and population (d).

**FIGURE A7:**
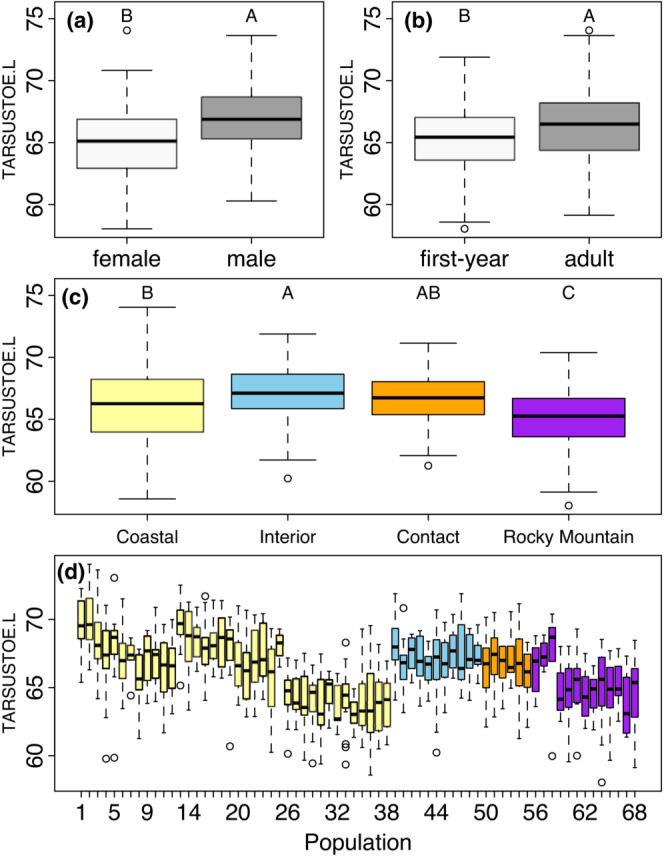
Box plots of tarsus plus middle toe measurements (mm) for comparisons by sex (a), age (b), morphotype (c), and population (d).

**FIGURE A8:**
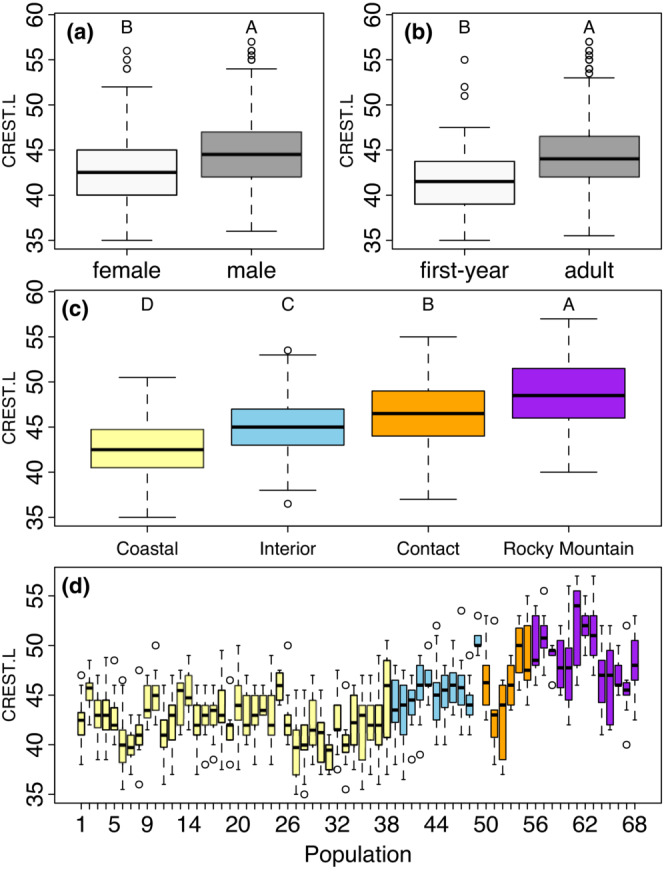
Box plots of crest length measurements (mm) for comparisons by sex (a), age (b), morphotype (c), and population (d).

**FIGURE A9:**
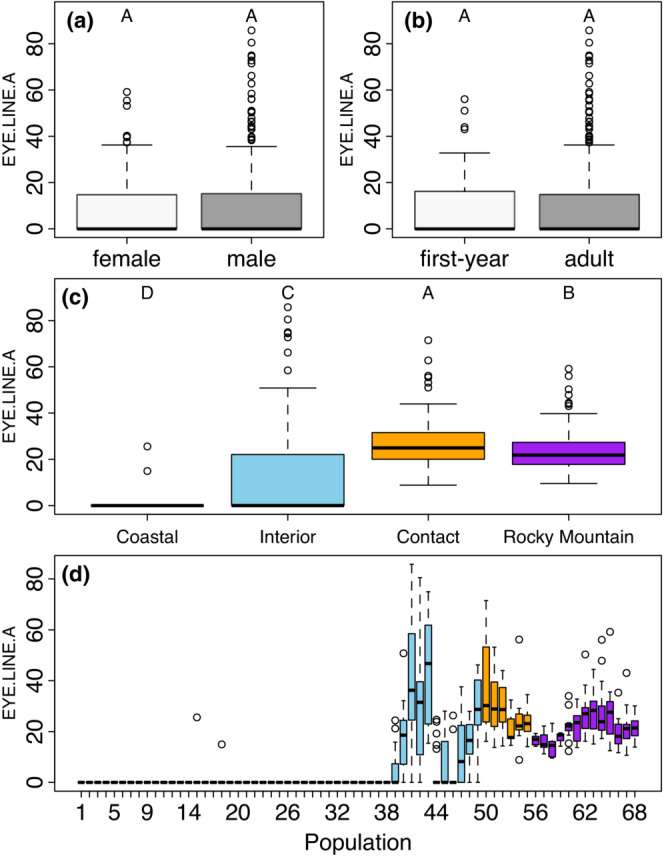
Box plots of eye line measurements (length × width, mm) for comparisons by sex (a), age (b), morphotype (c), and population (d).

**FIGURE A10:**
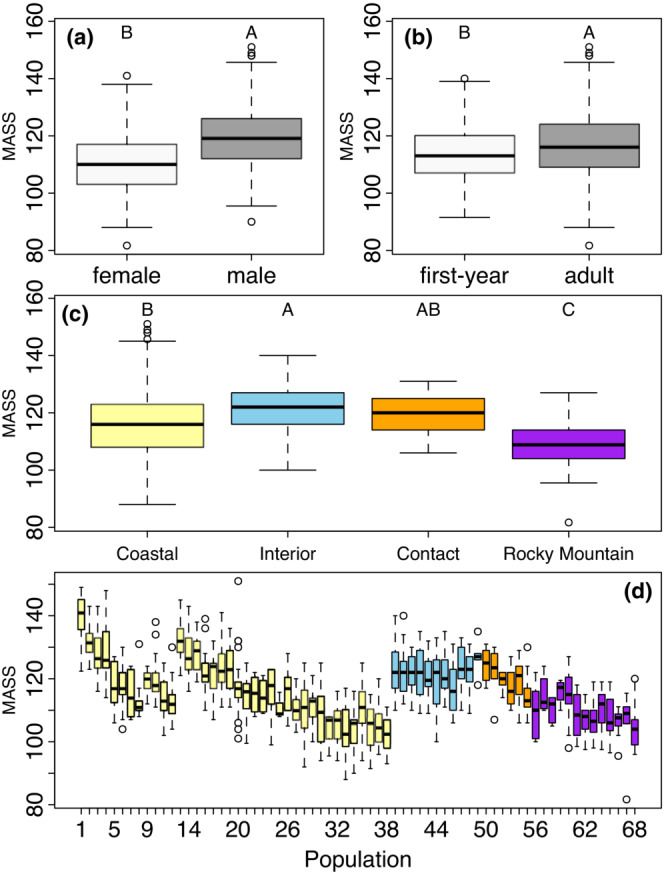
Box plots of body mass (grams) for comparisons by sex (a), age (b), morphotype (c), and population (d).

**FIGURE A11:**
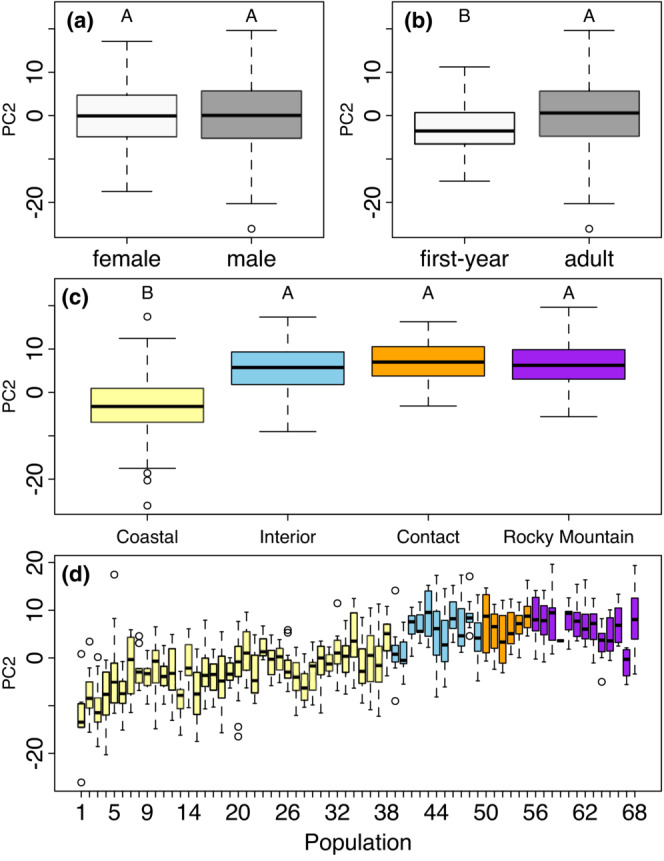
Box plots of PC2 values for morphological comparisons by sex (a), age (b), morphotype (c), and population (d).

More details emerged when examining differences between each of the 68 populations analyzed in the linear mixed models (Figure 2d). The most striking pattern was in the Coastal morphotype, which showed two parallel north‐south clines in size (larger birds in the north) that corresponded to populations in the Coast Ranges (1–12) and Cascades‐Sierra Nevada (13–38), respectively. Steller's Jays from populations representing the Interior morphotype (39–49) were relatively large and similar in size to birds from the more northern Coastal populations. Rocky Mountain birds (56–68) also showed a gradual trend toward smaller size in the south, but overall, they were similar morphologically to Steller's Jays from the central and southern part of the Coastal morphotype range. Morphological differences in individual traits and in PC2 all showed significant differences between at least two morphotypes (Table A5 and Figures A2, A3, A4, A5, A6, A7, A8, A9, A10, A11).

**FIGURE 3:**
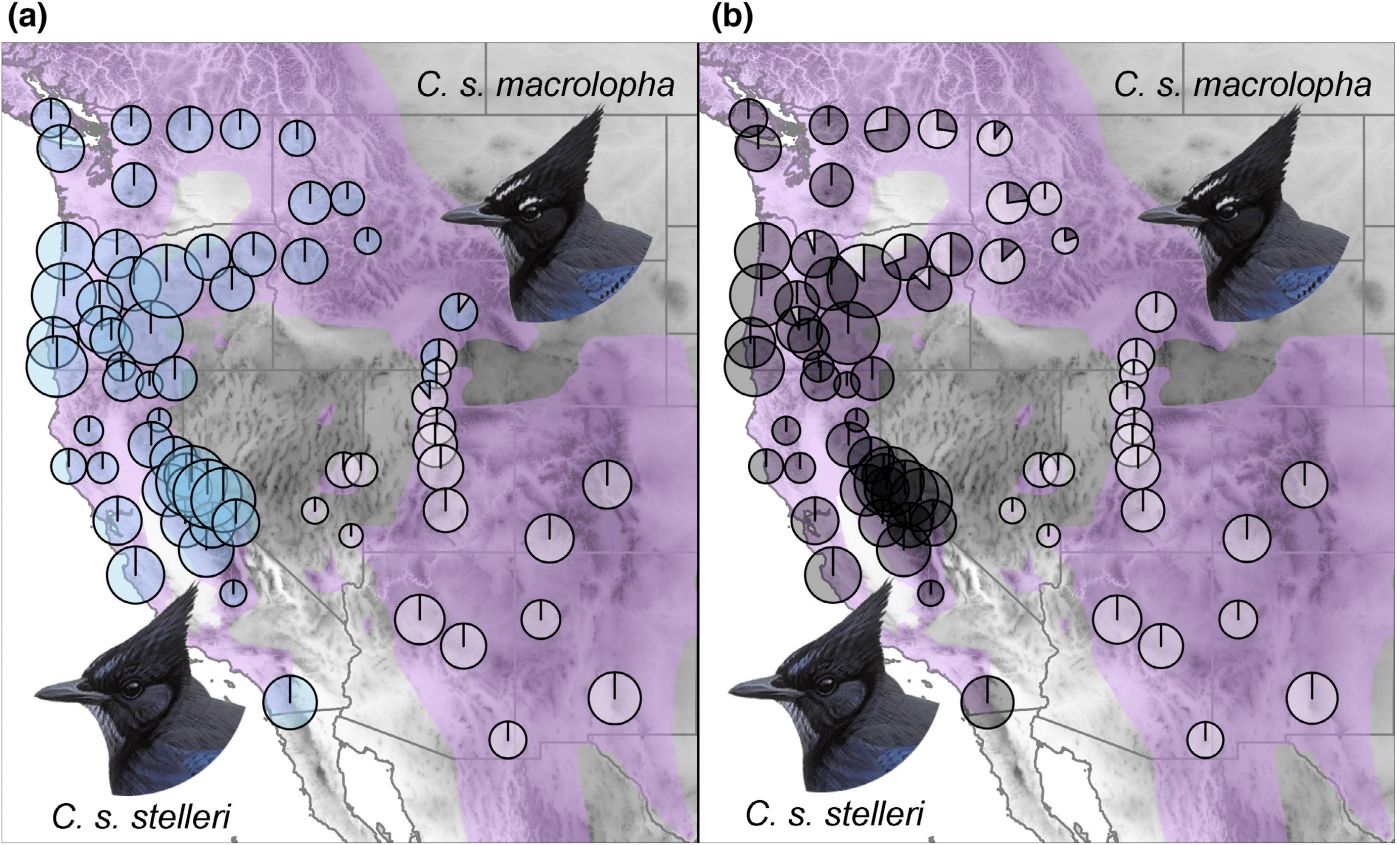
Geographic variation in the frequency of (a) blue or white frontal streaks and (b) white supercilium among populations of Steller's Jays. Size of each circle is proportional to the number of individuals sampled from each population (as in Figure 1). For panel (a), entirely blue or white circles indicate that all birds in that population had blue or white streaking, respectively. For panel (b), entirely black or white circles indicate that all birds lacked or had a white supercilium, respectively. Illustrations are reproduced from https://birdsoftheworld.org with permission from Lynx Edicions.

The two facial traits examined (frontal streak color, superciliary line) showed strong geographic patterning among populations that corresponds to the three morphotypes (Figure 3). Frontal streaking was scored as blue for all individuals from Coastal and Interior morphotypes (Figure 3a), with a transition from blue to white across the contact zone (populations 50–55, Figure 1) to Rocky Mountain populations. With few exceptions, Coastal birds lacked a superciliary line while all Rocky Mountain birds had a prominent line (Figure 3b; Figure A9). For Interior populations, the prominence of a superciliary line changed from low frequency in the west to higher frequency in the east. All birds in the contact zone had a superciliary line.

**FIGURE 4:**
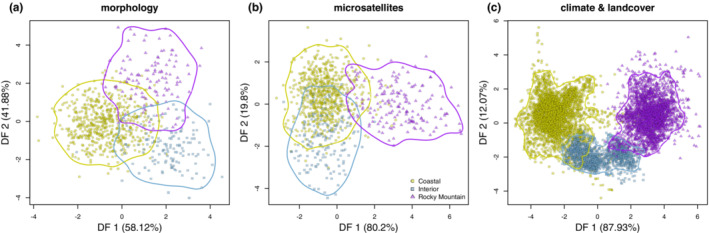
Scatterplots of discriminant function scores and associated classifications by Steller's Jay morphotype for different traits: (a) Discriminant Function Analysis (DFA) based on continuous morphological characters. (b) Discriminant Analysis of Principal Components (DAPC) based on 12 nuclear microsatellite loci. (c) DFA based on the reduced set of 13 climate predictors employed for the SDM analyses in addition to a landcover predictor representing closed habitat types. Each point represents an individual whose morphotype is defined according to subspecies and/or sampling locality. Contour lines encompass 95% of the data for each morphotype. The percent variance explained by each discriminant axis is provided in the axis labels.

Discriminant Function Analysis of the morphological data showed strong separation among the three morphotypes, and cross‐validation led to high accuracy in predicting the correct morphotype (Table 3, Figure 4a). Specifically, 95.9% of Coastal Steller's Jays, 84.8% of Interior jays, and 80.0% of Rocky Mountain jays were classified correctly (Table 3). The 80%–96% success in classification indicates that morphological variation is partitioned geographically in Steller's Jays.

**TABLE 3:** Percentage of Steller's Jay individuals classified into different morphotypes by Discriminant Function Analysis (DFA, morphology and environmental) and Discriminant Analysis of Principal Components (DAPC, microsatellites).

Actual	Predicted		
	Coastal (%)	Interior (%)	Rocky (%)
*Morphology*			
Coastal	**95.9**	1.9	2.2
Interior	14.5	**84.8**	0.7
Rocky Mtn.	14.8	5.2	**80.0**
*Microsatellites*			
Coastal	**94.4**	4.3	1.3
Interior	36.7	**63.3**	0.0
Rocky Mtn.	9.7	0.0	**90.3**
*Climate + landcover*			
Coastal	**94.5**	4.6	0.9
Interior	6.2	**91.8**	2.0
Rocky Mountain	0.0	2.7	**97.3**

*Note*: Contact zone samples (populations 50–55) were excluded from these analyses. Correct classifications are highlighted in bold.

### 3.2 Mitochondrial DNA variation

**FIGURE 5:**
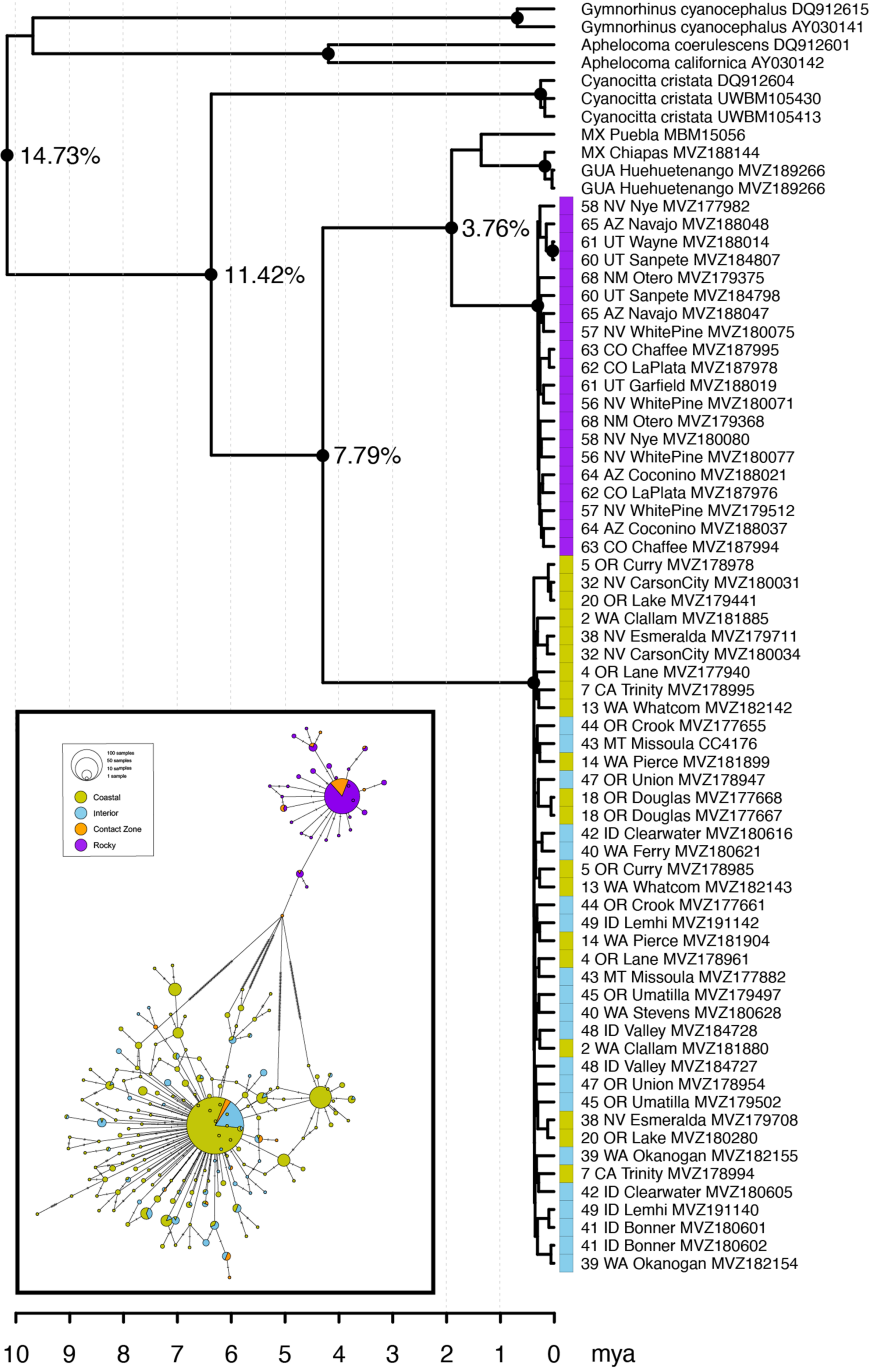
Phylogeny of Steller's Jay populations and related taxa inferred from the mitochondrial gene region ND2. Nodes with black circles received greater than 95% posterior probability from the Bayesian BEAST analysis and over 70% bootstrap support from the RAxML maximum Likelihood analysis. The average uncorrected percentage of pairwise sequence divergence between various clades is shown, while the time scale in million years (mya) is shown at the bottom. The color at each tip corresponds to that individual's morphotype. Inset shows the haplotype network from ND2 alignments. The size of each circle corresponds to the number of individuals that share a given haplotype, while the proportion of color within a circle indicates which morphotypes share a given haplotype. Unsampled haplotypes are shown with tick marks.

The ND2 phylogeny and haplotype networks showed congruent patterns of mitochondrial DNA variation within and between the three morphotypes (Figure 5). In both the BEAST and RAxML analyses, Steller's Jays form two distinct and well‐supported clades that separate Rocky Mountain populations from those in Coastal and Interior groups. The average uncorrected sequence divergence of 7.79% shows a deep split between these clades that dates to approximately 4.29 mya (95% highest posterior probability = 3.39–5.13). This split ranks among the highest relative divergence compared with other pairs of North American avian species examined in this study (ND2 range 0.0%–9.3%, Table 4). Another split within the Rocky Mountain clade (3.76% uncorrected sequence divergence) separates populations of Steller's Jays in the Rocky Mountains from those in central to southern Mexico and Central America. By contrast, we found no structure in either the ND2 phylogeny or haplotype network that distinguished Coastal and Interior morphotypes. Furthermore, the network analysis revealed a narrow geographic cluster of populations (50–55) with haplotypes from both the Coastal/Interior and Rocky Mountain clades, consistent with the contact zone samples identified in the morphological and microsatellite data; no populations outside of the contact zone had mixed ND2 haplotypes. Our sequence data suggest that the Steller's Jay diverged from its sister species the Blue Jay approximately 6.36 mya (95% highest posterior density = 5.20–7.46 mya).

**TABLE 4:** Percent uncorrected sequence divergence in ND2 between 30 species pairs of North American birds, for comparison with Coastal (including Interior) and Rocky Mountain morphotypes of Steller's Jay.

	Sister species 1	Sister species 2	Mean div (%)	Div range (%)
1	*Acanthis flammea*	*Acanthis hornemanni*	0.1	0.0–0.2
2	*Zonotrichia leucophrys*	*Zonotrichia atricapilla*	0.1	0.0–0.6
3	*Pica nuttalli*	*Pica hudsonia*	0.1	0.0–0.7
4	*Sphyrapicus ruber*	*Sphyrapicus nuchalis*	0.7	0.0–1.5
5	*Baeolophus bicolor*	*Baeolophus atricristatus*	0.8	0.8–0.9
6	*Anser caerulescens*	*Anser rossi*	0.9	0.9–0.9
7	*Junco hyemalis*	*Junco phaeonotus*	0.9	0.0–1.8
8	*Quiscalus major*	*Quiscalus mexicanus*	1.0	0.9–1.3
9	*Artemisiospiza belli*	*Artemisiospiza nevadensis*	1.0	0.6–2.4
10	*Setophaga townsendi*	*Setophaga occidentalis*	1.2	1.0–1.3
11	*Centrocercus urophasianus*	*Centrocercus minimus*	1.2	1.0–1.5
12	*Pipilo maculatus*	*Pipilo erythrophthalmus*	1.3	1.1–1.5
13	*Toxostoma cinereum*	*Toxostoma bendirei*	1.6	1.0–2.8
14	*Rallus longirostris*	*Rallus elegans*	1.7	0.7–2.7
15	*Ammodramus caudacutus*	*Ammodramus nelsoni*	2.0	2.0–2.0
16	*Catharus minimus*	*Catharus bicknelli*	2.3	2.1–2.5
17	*Vireo solitarius*	*Vireo cassinii*	2.5	2.1–2.9
18	*Aphelocoma woodhouseii*	*Aphelocoma californica*	3.7	3.5–4.1
19	*Baeolophus inornatus*	*Baeolophus ridgwayi*	4.2	4.2–4.2
20	*Lagopus mutus*	*Lagopus lagopus*	4.5	4.5–4.5
21	*Polioptila californica*	*Polioptila melanura*	4.7	3.9–7.2
22	*Passerina versicolor*	*Passerina ciris*	5.2	5.2–5.2
23	*Empidonax alnorum*	*Empidonax traillii*	5.3	5.0–5.7
24	*Sturnella magna*	*Sturnella neglecta*	5.5	4.7–6.3
25	*Passerina caerulea*	*Passerina amoena*	7.1	7.0–7.2
26	*Piranga ludoviciana*	*Piranga bidentata*	7.4	7.2–7.6
27	*Megascops kennicottii*	*Megascops asio*	7.5	7.0–7.9
28	*Limnodromus griseus*	*Limnodromus scolopaceus*	7.8	7.8–7.8
	***Cyanocitta stelleri*‐Coastal**	***Cyanocitta stelleri‐*Rocky Mtn.**	**7.8**	**7.3–8.3**
29	*Contopus sordidulus*	*Contopus virens*	7.9	7.4–8.8
30	*Colinus cristatus*	*Colinus virginianus*	8.1	7.3–9.3

Population genetic measures (Table 5) showed a total of 288 unique ND2 haplotypes. Populations representing the Coastal morphotype had the largest sampling (*N =* 683) and the most unique haplotypes (*N =* 198). Although Interior and Rocky Mountain morphotypes had equivalent sampling, Interior populations had approximately 1.6 times the number of unique haplotypes. Similarly, nucleotide diversity and both genetic diversity estimates (θ) were highest in Coastal populations, intermediate in the Interior, and lowest in Rocky Mountain populations. Fu's (1997) *F*_S_ and Tajima's (1989) *D* statistics were significantly negative for all morphotypes, indicating an excess of segregating sites relative to haplotype divergence.

**TABLE 5:** Population genetic measures for three morphotypes of Steller's Jay based on ND2 sequences (*N =* 1019). We excluded 61 individuals from six populations in the contact zone for these analyses.

Morphotype	*N*	Unique haplotypes	Nucleotide diversity	*θ*_S_	*θ*_π_	*F*_S_	*D*
Coastal	683	198	0.002041 ± 0.001258	24.78 ± 4.77	2.12 ± 1.31	−26.22*	−2.66*
Interior	167	55	0.001631 ± 0.001062	10.19 ± 2.59	1.70 ± 1.11	−27.81*	−2.54*
Rocky Mtn.	169	35	0.000982 ± 0.000732	6.49 ± 1.76	1.02 ± 0.76	−29.15*	−2.48*

*Note*: Asterisks indicate Fu's (1997) *F*_S_ and Tajima's (1989) *D* values significant at *p* < .0001.

### 3.3 Microsatellite variation and genetic connectivity

We successfully scored all 12 nuclear microsatellite loci for 1069 of the 1075 Steller's Jay individuals analyzed. One individual was successful for only 10 loci, and five individuals were successful for 11 loci (Table S1). All 12 loci were polymorphic across each of the 68 populations included in the analyses (Table S6). The highest number of alleles at any locus was 16, found at locus Cst7 in populations 37 (Mineral County, Nevada) and 44 (Crook County, Oregon); the lowest number of alleles within a population was three. Allelic richness ranged from 2.82–8.64 and observed heterozygosities (H_O_) varied from 0.25 to 1.0. When populations were grouped by morphotype (Table A6), the number of alleles ranged from 8–22 and allelic richness varied from 7.553–21.988. Overall, allelic richness was most variable in Interior populations (7.976–21.988) followed by Coastal (7.78–17.591) and Rocky Mountain (8.988–16.994) populations. Observed heterozygosities (*H*_O_) varied from 0.72–0.92 across the three morphotypes.

The only significant departure from Hardy–Weinberg equilibrium occurred in locus Cst58 in Rocky Mountain populations after Holm–Bonferroni sequential correction for multiple comparisons (adjusted *p‐*value < .001, Table A6). Pairwise comparison tests for linkage disequilibrium showed that even though there was evidence of significantly linked loci‐pairs in each of the three main population groups, there was no consistent pattern in loci linkages throughout the whole dataset (Table S7). Therefore, we retained all loci in the dataset for further analyses.

**FIGURE 6:**
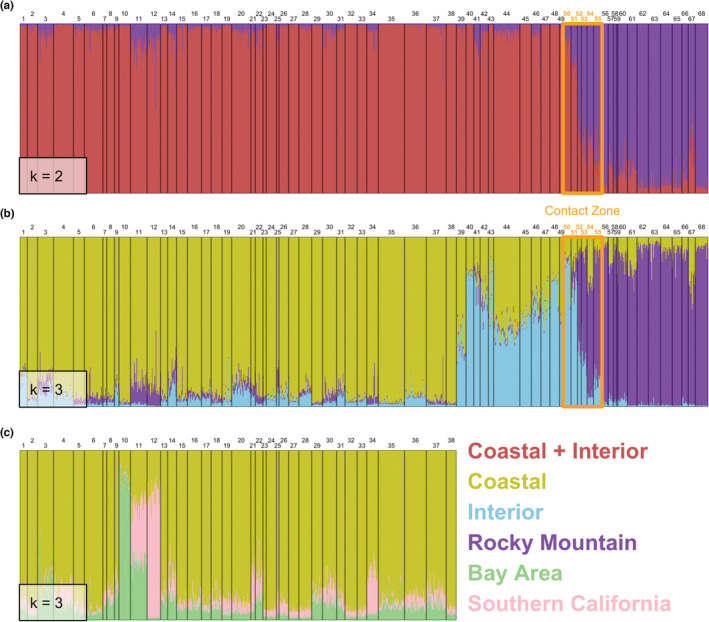
STRUCTURE results based on 12 nuclear microsatellite loci for 68 populations and 1075 individuals of Steller's Jay. Numbers correspond to populations in Figure 1 and Table S1, which were ordered to correspond with geography and ecomorphological phenotypes and assigned to clusters based on the *Q* scores from the STRUCTURE output. (a) Split of all populations into two broad groups (*K* = 2): Coastal plus Interior morphotype and Rocky Mountain morphotype. The orange rectangle outlines the contact zone between the two groups, which is delineated based on transitions in phenotype (especially color of frontal streaks), mtDNA haplotypes, and microsatellite *Q* scores. (b) Further subdivision of all populations into Coastal, Interior, and Rocky Mountain groups (*K* = 3), with the contact zone transition indicated. (c) Coastal‐only populations (1–38) showing three genetically distinct clusters (*K* = 3) separating San Francisco Bay Area (Contra Costa, 10) and southern California (San Diego, 12) populations; Monterey (11) shares alleles with both of those populations.

Clustering analysis of the microsatellite data for all 68 populations using STRUCTURE showed an optimal value of *K* = 2 using the using Delta *K* (Δ*K*) method (Evanno et al., 2005; Table A7, Figure A1). This is manifested as a sharp split of Coastal plus Interior versus Rocky Mountain populations (Figure 6a), which is congruent with analyses of the ND2 sequence data. Membership coefficients from this analysis reveal a sharp transition from one group to the other across populations 50–55 in the putative contact zone.

**FIGURE A12:**
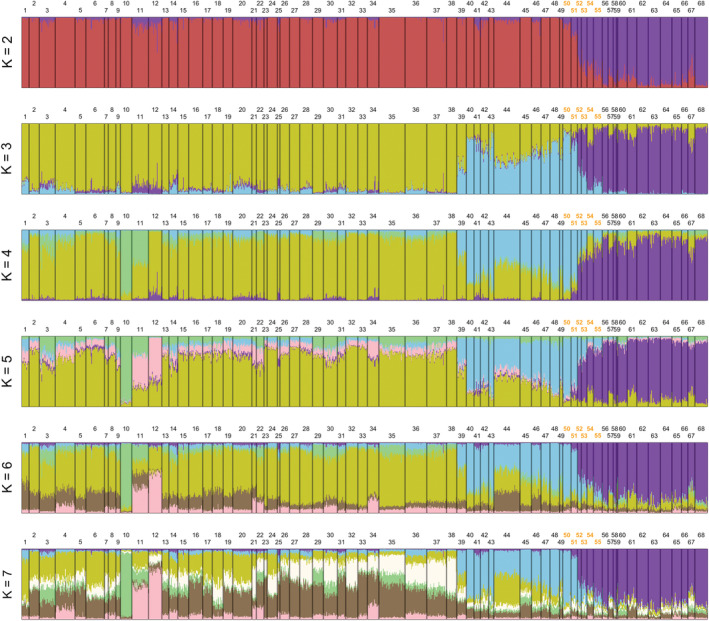
STRUCTURE plots of microsatellite data (*K* = 2 through *K* = 7) for all 68 populations of Steller's Jays sampled in this study. Numbers correspond to populations in Figure 1 and Table S1, which were ordered to correspond with geography and ecomorphological phenotypes and assigned to clusters based on the Q scores from the STRUCTURE output. Colors of the different genetic clusters are the same as in Figure 6.

While Δ*K* indicated an optimal *K* = 2 for the entire dataset, likelihood Ln *P*(*K*) values showed support for a higher *K* = 4 across all populations (Table A7, Figure A1) that separated the three morphotypes (Figure 6b) and revealed some structure within Coastal populations (see STRUCTURE runs up to *K* = 7, Figure A12). All plots show more introgression from Coastal to Interior regions than in the opposite direction. As with *K* = 2, the sharp transition from Interior to Rocky Mountain morphotypes is maintained across the contact zone.

These results prompted us to further examine the genetic substructure within each of the Coastal, Interior, and Rocky Mountain populations separately. Within the Coastal morphotype, both Δ*K* and Ln *P*(*K*) had an optimal value of *K* = 3 that distinguished San Francisco Bay Area (Contra Costa, 10) and southern California (San Diego, 12) populations from each other and the remaining Coastal populations except for Monterey (11), which shared microsatellite alleles with both Contra Costa and San Diego (Figure 6c; Figure A12). Populations in coastal central California have been recognized as a separate subspecies (*C. s. carbonacea*) that occurs only from the San Francisco Bay region south to Monterey Bay (Grinnell, 1900; Grinnell & Miller, 1944). Within the Rocky Mountain morphotype, our dataset showed an optimal value of *K* = 2 using both methods. Although STRUCTURE plots (Figure 6; Figure A12) do not indicate two clusters within that morphotype, birds from southern New Mexico (Otero, population 68) appear to have some unique alleles relative to all other Rocky Mountain individuals. One possible explanation is introgression from Steller's Jays to the south in Mexico (e.g., Sierra Madre Occidental); an additional study that includes populations in Mexico and Central America is in progress (McCormack et al. unpublished). Finally, we did not find any evidence of clustering within the Interior morphotype (mean likelihood value of *K* = 1).

Discriminant Analysis of Principal Components (DAPC) based on the 12 microsatellite loci showed strong discrimination of the three morphotypes along the two discriminant function (DF) axes (Table 3, Figure 4b). The first DF axis (80.2% of the variance) separated Rocky Mountain from Coastal plus Interior populations. The second DF axis (19.8% of the variance) showed discrimination between Coastal and Interior populations. These results are consistent with those found in the STRUCTURE analyses for *K* = 3. Individuals from both Coastal and Rocky Mountain populations were predicted to be in the correct group with high accuracy (94.4% and 90.3% correct classification, respectively; Table 3). Of the individuals that were misclassified, 4.3% of Coastal morphotype birds were predicted to be in the Interior group and 1.3% were predicted to be in the Rocky Mountain group; conversely, 19.7% of Rocky Mountain individuals were predicted to be in the Coastal group and none in the Interior group. The prediction was much lower for Interior populations (63.3% correct), where all misclassified individuals (36.7%) were predicted to be in the Coastal group (Table 3).

**FIGURE 7:**
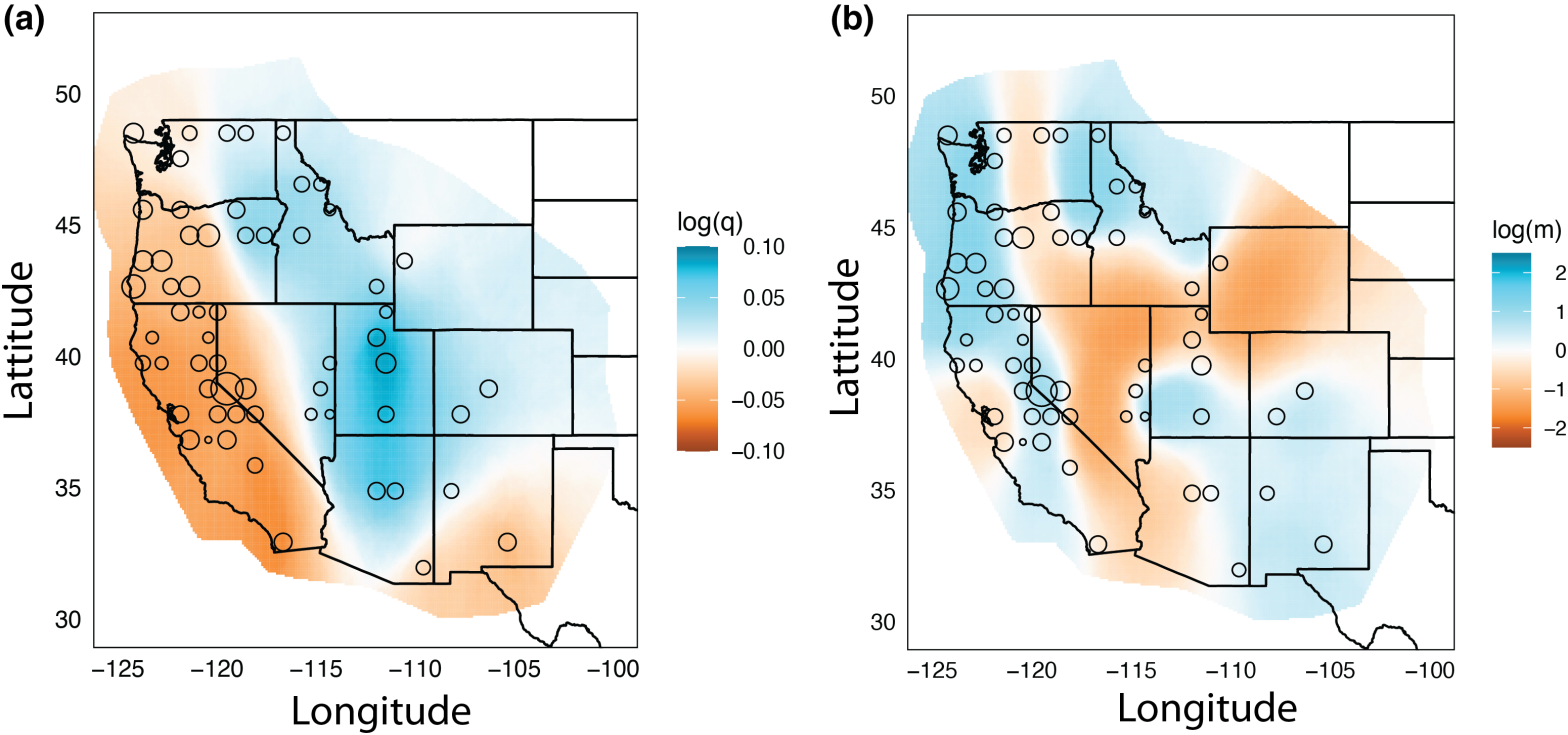
Estimated effective migration surfaces (EEMS) for Steller's Jays in the western United States, showing (a) nuclear genetic diversity and (b) effective migration rates; blue colors indicate higher genetic diversity and migration rates while orange colors indicate lower genetic diversity and migration rates, respectively. The polygon outlining our sampling localities represents the geographic extent of the analysis, within which populations were assigned to one of 500 demes represented by nodes. The size of each circle is proportional to the number of individuals assigned to a given node.

The map of nuclear genetic diversity for Steller's Jays (Figure 7a) showed the highest levels in the central to southern Rocky Mountains (e.g., Utah and northern Arizona). Overall, diversity was higher throughout the Rocky Mountain and Interior populations compared with the Coastal region. The lowest diversity was found in central coastal and southwestern California. Diversity also was relatively low in southeastern Arizona and southern New Mexico. Estimating effective migration surfaces (Figure 7b) showed several areas of reduced gene flow: (1) contact zone between Pacific Northwest and Interior populations in central Washington and Oregon; (2) the Great Basin and southwestern desert regions that separate Coastal and Rocky Mountain populations; (3) contact zone that separates Interior and Rocky Mountain populations where the Columbia Plateau, Great Basin, and Rocky Mountain system meet (southeastern Idaho, southwestern Wyoming, and northeastern Utah); and (4) central California relative to the rest of the Coastal populations.

The nuclear genetic diversity results are consistent with the private allele analysis (Table 6), which showed that a much higher percentage of Rocky Mountain populations had private alleles (30.8%) compared with Coastal (10.5%) and Interior (9.1%) populations; an even higher percentage of populations with private alleles (50.0%) occurred in the contact zone. Although the number of populations sampled was approximately three times higher for Coastal versus Rocky Mountain morphotypes, an equivalent number and proportion of private alleles were found in the two regions (5.3% vs. 5.6% of all alleles genotyped, respectively). The presence of private alleles was lowest in populations of the Interior morphotype (1.4% of alleles genotyped).

**TABLE 6:** Private microsatellite alleles across all populations, grouped by morphotype

Morphotype	Total # of populations	# (%) of populations with private alleles	Total # alleles genotyped	# (%) of private alleles	Average frequency of private alleles
Coastal	38	4 (10.5%)	152	8 (5.3%)	0.035
Interior	11	1 (9.1%)	140	2 (1.4%)	0.050
Contact	6	3 (50.0%)	140	7 (5.0%)	0.059
Rocky Mtn.	13	4 (30.8%)	144	8 (5.6%)	0.034

### 3.4 Cline analyses

**FIGURE 8:**
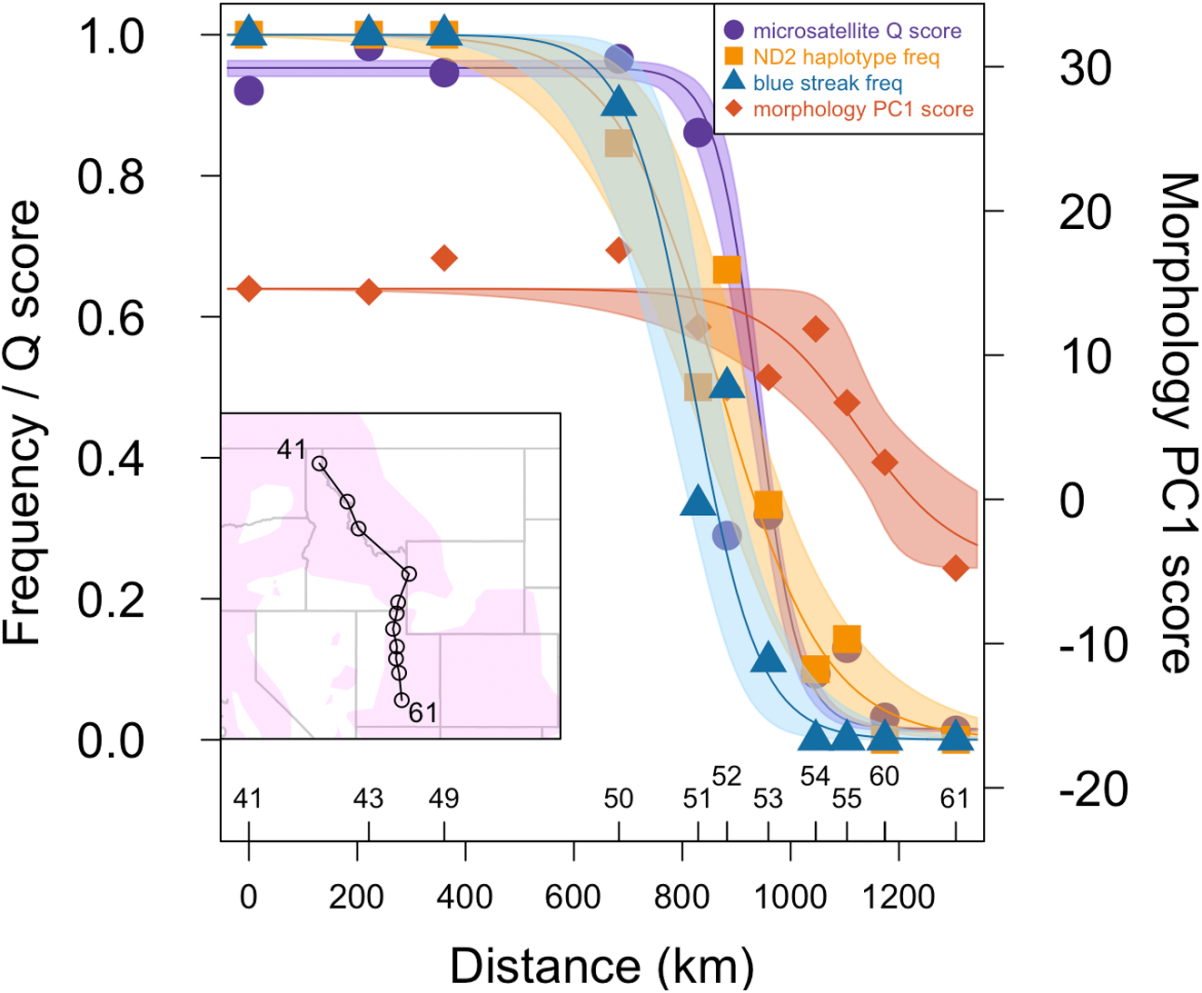
Cline analysis (HZAR) for microsatellite Q scores (purple circles), mtDNA (ND2) haplotype frequencies (orange squares), blue frontal streak frequencies (blue triangles), and morphology PC1 (red diamonds). Lines indicate the maximum likelihood cline model fitted for each trait. Shaded areas represent 95% credible intervals, and symbols represent the mean values for each sampling location. Inset map shows the sampling transect, with each circle representing a population. Population numbers correspond to those in Figure 1 and are plotted above the *x*‐axis to show the location and distance of symbols on each cline. Note that the cline for morphology PC1 is on a different scale and axis than the other three traits.

HZAR fits to the data showed strong and geographically coincident clines (Figure 8) in ND2 haplotype frequencies, blue frontal streak frequencies, and microsatellite Q scores between populations 50–55 that span 421 km. The microsatellite data showed the steepest cline, which was offset slightly to the south compared with the other two traits; this break occurred between populations 51 and 53, which are located 129.7 km apart. Although morphological PC1 scores also showed a general cline in this region, the curve was much shallower and occurred over a larger geographic distance to the south (populations 53–60, 215 km). A comparison of the different HZAR models and associated values is given in Table A8.

### 3.5 Environmental variation

Collinearity reduction of climatic variables decreased the set of predictors from 33 to 12. When landcover variables were included, the initial set of 42 predictors (33 climates plus 9 landcovers) was reduced to 22. A list of predictor variables is given in Table A9. Feature classes and smoothing parameters for MaxEnt, optimized via AICc model selection, can be found in Table A10.

**FIGURE 9:**
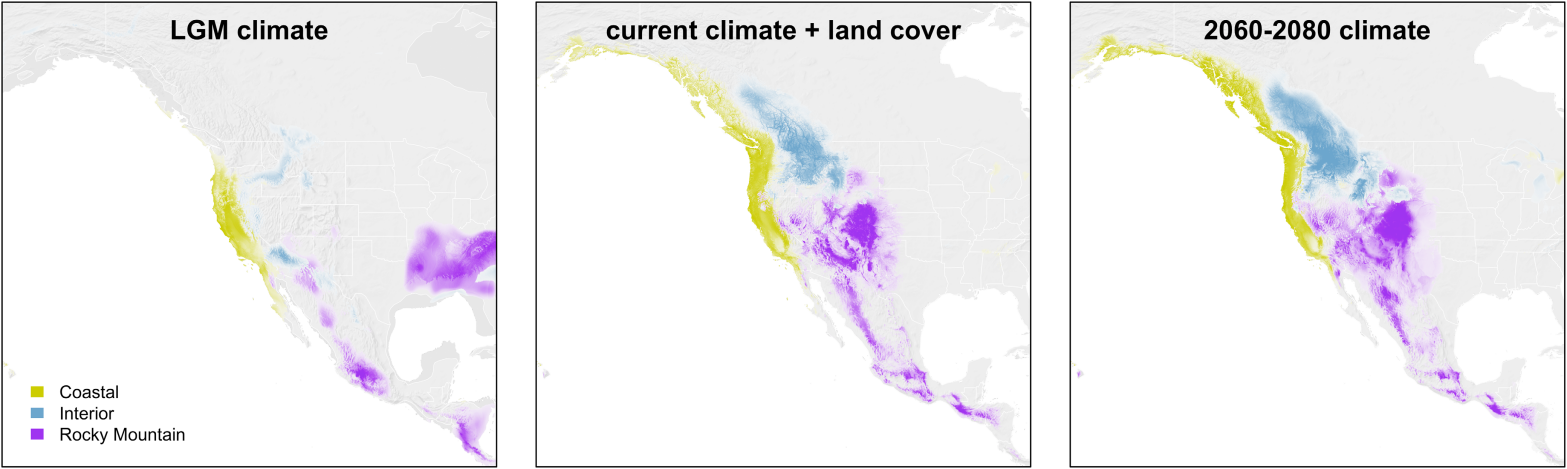
Maps showing the projected distribution of Steller's Jay morphotypes based on environmental variables. Projections for the Last Glacial Maximum (LGM) and future (2060–2080) climate scenarios were based solely on bioclimatic variables. Present‐day distribution models were based on current climate and landcover data.

Species distribution models for the three morphotypes (Figure 9) showed strong geographic patterning. The model based on the current climate and landcover closely matched present‐day distributions and separated the morphotypes into corresponding Coastal, Interior, and Rocky Mountain ecoregions. Distribution of the Coastal morphotype extended from southeastern Alaska through the Pacific Northwest, Coast Ranges, and Cascades‐Sierra Nevada to southern California. An ecological break separates that range from the Interior morphotype distribution that occupies interior western Canada to the northern Rocky Mountains. Another break separates Interior and Rocky Mountain morphotypes, with the latter having the highest suitability in the Rocky Mountains but also extending into the Great Basin and from the Southwestern United States through Mexico to northern Central America.

Projections to the Last Glacial Maximum (LGM, Figure 9) showed the Coastal morphotype to have the most stable distribution, with the main difference being an absence of suitable habitat from northwestern Washington to southeastern Alaska. The interior region of western North America showed little suitability for Steller's Jays during the LGM, with one pocket occurring in the Columbia Plateau and northern Rockies and another in the Southwestern United States. Finally, the LGM distribution for the Rocky Mountain morphotype appears to have been pushed southward into disjunct populations in Mexico and Central America, as well as eastward into the southeastern United States (a range currently occupied by the Blue Jay, sister species to the Steller's Jay).

Future climate projections (2060–2080, Figure 9) revealed little change in morphotype distributions from their current respective ranges. The biggest difference was in the Interior, with a more suitable future habitat predicted for that morphotype (especially toward the Coastal range). The model also projected the expansion of suitable habitat for the Rocky Mountain morphotype in the Great Basin.

**FIGURE 10:**
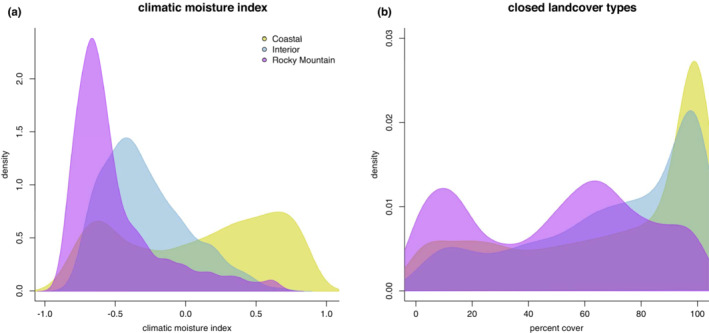
Density plots of Steller's Jay morphotype occurrence records relative to Climatic Moisture Index and percent landcover. Percent cover indicates the percent of each grid cell that is occupied by closed habitat types. Coastal and Interior morphotypes appear to preferentially inhabit regions with more closed habitats compared with the Rocky Mountain morphotype.

**FIGURE A13:**
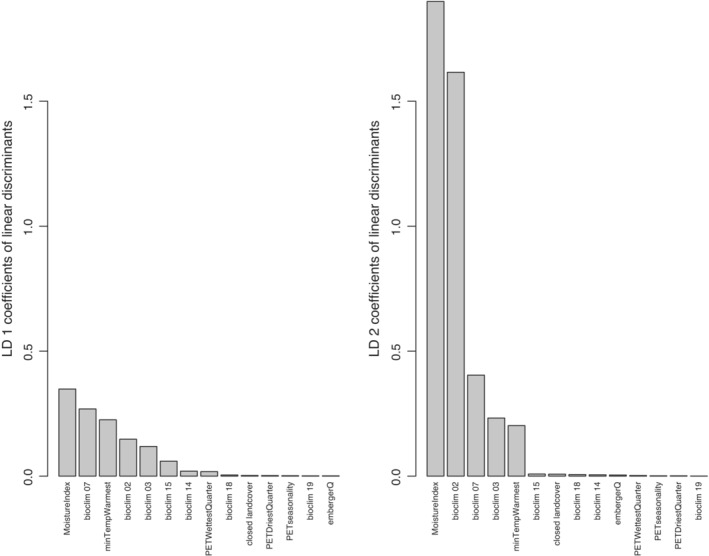
Absolute values of DFA loadings for 13 climate and 1 landcover variables. See Table A8 for further description of the predictors.

**FIGURE A14:**
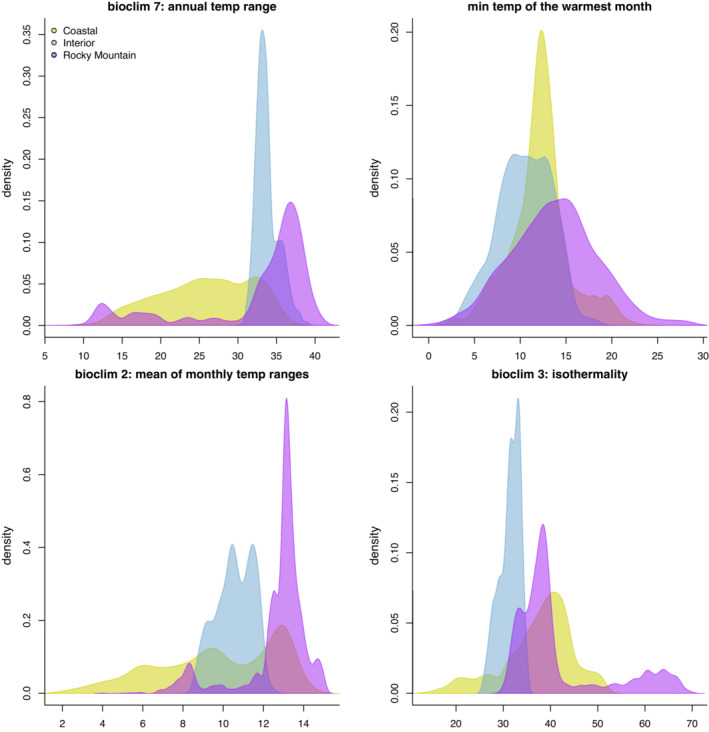
Density plots of four climate predictors with strong contributions to the DFA, based on the geographic occurrence records for each morphotype.

The reduced set of variables for the Discriminant Function Analysis (DFA) consisted of 13 climatic predictors plus closed habitat landcover. Variables that contributed most to the discrimination included Climate Moisture Index (CMI), mean monthly temperature range (BIO2), isothermality (BIO3), annual temperature range (BIO7), and minimum temperature of the warmest month (Figure A13). Examination of density plots by morphotype for these five variables (Figure 10; Figure A14) showed clear environmental differences between groups, with Rocky Mountain birds occurring most commonly in drier areas with a higher temperature range. Furthermore, examination of morphotypes in relation to percent landcover showed that Rocky Mountain birds tend to occur more in open habitats (lower percent cover) compared with Coastal and Interior birds (Figure 10). The DFA plot (Figure 4c) showed strong separation of morphotypes based on climate and landcover, with over 90% of the occurrence records classified into the correct morphotype (94.5% for Coastal, 91.8% for Interior, 97.3% for Rocky Mountain, Table 3).

## 4 DISCUSSION

### 4.1 Phenotypic and genetic variation

Steller's Jays of the western United States show strong geographic structuring in both phenotype and genotype. Further, the observed patterns are congruent with morphotype groupings and, to some extent, with the current subspecies classification (Clements et al., 2021). The Coastal morphotype showed the strongest discrimination in morphology, with 96% of individuals correctly classified (Table 3), and all three morphotypes were distinguished on the basis of plumage traits (Coastal—no eye line, blue frontal streaks; Interior—white eye line, blue frontal streaks; Rocky Mountains—white eye line, white frontal streaks). Within the contact zone between Interior and Rocky Mountain morphotypes, Steller's Jays were most similar morphologically to Interior birds but showed mixed traits in head phenotype patterns; all contact zone individuals had a white superciliary line typical of the Rocky Mountain morphotype, while the color of the frontal streaks gradually changed from blue (north) to white (south) across the contact zone (Figure 3). Another finding from our study was a trend toward larger body size at higher latitudes, especially among populations in the Coast Ranges and Cascade‐Sierra Nevada Mountains (Figure 2). A similar, albeit weaker, trend was observed among Rocky Mountain populations. Walker et al. (2020) likewise described a general increase in body size from south to north and reported a high degree of intermediacy between subspecies of Steller's Jays. Latitudinal trends in body size and their association with temperature (i.e., “Bergmann's rule”, Bergmann, 1847) have been found to occur on a global scale in birds (Ashton, 2002), and may result from both phenotypic plasticity and genetically‐based adaptations (Stillwell, 2010). Bay (2002) showed that morphological differentiation in Steller's Jays is correlated with habitat and appears to be adaptive to local environments. Studies of other phenotypically variable taxa (e.g., *Junco hyemalis*) have shown that local adaptation driven by environment can promote rapid differentiation (Friis et al., 2018).

Genetic data from both mitochondrial DNA sequences and microsatellites are congruent with the phenotypic breaks between morphotypes. DNA sequences showed a primary subdivision separating Rocky Mountain populations from Coastal plus Interior populations, with no pattern observed within the Coastal/Interior mtDNA lineage. Our comparison of uncorrected ND2 divergences between North American avian species pairs shows that Coastal/Interior versus Rocky Mountain Steller's Jays are equally or more divergent than many pairs of well‐established avian biological species. Microsatellites supported the divergence of Coastal versus Rocky Mountain populations and showed further subdivision that separated Coastal from Interior populations and central California birds from all other Coastal populations. In contrast with a prior suggestion of substantial gene flow between subspecies (Walker et al., 2020), Steller's Jays exhibit higher levels of population structure than other corvid species (Burg et al., 2005) and show reduced gene flow (this study) between: (1) Pacific Northwest and Interior populations in central Washington and Oregon; (2) Coastal and Rocky Mountain populations across the Great Basin and southwestern desert regions; (3) Interior and Rocky Mountain populations across the Great Basin to the Great Divide Basin in Wyoming; and (4) central California populations relative to the rest of the Coastal morphotype.

Our finding of differentiation in central Coastal California provides evidence of the historical isolation of the Steller's Jay subspecies *carbonacea* described over a century ago (Fisher, 1902; Grinnell, 1900). This subspecies was named on the basis of size and color characters intermediate between *stelleri* and *frontalis*, and its distribution was described originally as coastal Oregon and California from the Columbia River south to Monterey County (west of the Cascades; Grinnell, 1900; Fisher, 1902). Later, its range was restricted to central Coastal California based on examination of newly collected specimens (including those in fresh plumage) from northwestern California (Maillard, 1922). Maillard (1922) noted that the central coastal area occupied by *carbonacea* “was supposed at one time to be either an island or a group of islands not widely separated,” and hypothesized that Steller's Jays spread from the interior mountains of California toward the coast where they diverged in isolation as an insular form. Support for this hypothesis comes from other taxa that show similar genetic and/or phenotypic divergence in central coastal California. For example, the songbird subspecies *Junco hyemalis pinosus* that breeds in this area is differentiated across its genome from other junco taxa (Friis et al., 2022), and this geographic region is a hotspot of mammalian (Davis et al., 2008) and herpetological (Rissler et al., 2006) diversification as well as plant endemism (Stebbins & Major, 1965). Extensive sampling and whole genome sequencing across diverse taxa (including Steller's Jays) for the California Conservation Genomics Project (Shaffer et al., 2022) will shed further light on lineage diversification in central coastal California as well as other regions and habitats in the state.

The divergence between Coastal, Interior, and Rocky Mountain populations of Steller's Jays in mtDNA and microsatellites is geographically coincident with splits between other montane avian taxa. Barrowclough et al. (2004) found three lineages of Blue Grouse sensu *lato* that have distributions similar to those of the three Steller's Jay morphotypes. These include a western clade found along the Coastal and Sierra Nevada‐Cascade ranges from western British Columbia to California (currently *D. fuliginosus*, Sooty Grouse, range similar to Coastal Steller's Jay), a southern clade found in the Rocky Mountains from eastern Nevada and northern Utah through the southern Rocky Mountains to Arizona and New Mexico (currently *D. obscurus*, Dusky Grouse, range similar to Rocky Mountain Steller's Jay), and a previously unrecognized northern clade in the northern Rocky Mountains and Columbia Plateau from British Columbia to southern Idaho (currently conspecific with *D. obscurus*, range similar to Interior Steller's Jay). The northern and southern Rocky Mountain clades of grouse meet in the same general area as Interior and Rocky Mountain morphotypes of Steller's Jay, although a detailed study of that contact zone is lacking. Other examples of western North American bird species that occupy coniferous forests and show three geographically congruent divisions include Hairy Woodpecker (*Dryobates villosus*, Klicka et al., 2011), Canada Jay (*Perisoreus canadensis*, van Els et al., 2012), and White‐breasted Nuthatch (*Sitta carolinensis*, Walstrom et al., 2012). A number of additional co‐distributed vertebrate species show west–east splits between Pacific (including Cascade‐Sierra Nevada) and Intermountain/Rocky Mountain populations, e.g., Mountain Chickadee (*Poecile gambeli*, Spellman et al., 2007), Brown Creeper (*Certhia americana*, Manthey et al., 2011), Swainson's Thrush (*Catharus ustulatus*, Ruegg, 2007), northern flying squirrel (*Glaucomys sabrinus* and the recently described *G. oregonensis*, Arbogast et al., 2017), Douglas and red tree squirrel (*Tamiasciurus douglasii* and *T. hudsonicus*, Arbogast et al., 2001), and Western Rattlesnake sensu *lato* (*Crotalus viridis*, Pook et al., 2000).

### 4.2 Ecomorphological associations

Results from this study integrating morphology and environment on a broad geographic scale, combined with evidence that birds in similar habitats (e.g., ponderosa pine) have similar morphologies in different geographic regions (Bay, 2002), provide strong evidence of ecomorphological associations in Steller's Jays. Discriminant function analysis and species distribution models for Steller's Jay occurrence records showed that the three morphotypes occur in distinct ecoregions based on both climate and landcover, and that 92%–97% of the records were correctly classified to morphotypes according to these ecological variables. Furthermore, occurrence density plots based on climate and percent landcover showed the Rocky Mountain morphotype to be more common in areas of lower moisture (Climatic Moisture Index), higher minimum temperature of the warmest month, higher monthly and annual temperature ranges (BIO2 and BIO7), higher isothermality (BIO3), and lower percent landcover compared with the other morphotypes. Coastal Steller's Jays occur in areas with the highest moisture and lowest temperature range, while Interior populations are intermediate in their climatic affinity. There did not appear to be any difference between Coastal and Interior populations in their occurrence relative to percent landcover, with both morphotypes being most common in areas of high cover.

Brown (1963a) suggested that dense vegetation should reduce the frequency of encounters through visual obstruction and that Steller's Jays are more likely to encounter conspecifics in open, drier habitats, thus leading to selection for more conspicuous crests and facial patterns used as visual cues (i.e., white frontal streaks, bright superciliary line, and longer crest as in the Rocky Mountain morphotype). Although we do not have behavioral data, the ecomorphological variation we observed is consistent with this suggestion. Specifically, Rocky Mountain Steller's Jays have the most prominent visual traits and occur in the driest, most open habitat. Studies of the association between plumage pattern and habitat in birds have revealed mixed results. Somveille et al. (2016) found no evidence globally for an association between habitat and plumage across more than 2700 bird species. However, other studies have shown that signaling conditions in different habitats (open versus closed) with varying light environments appear to be a major factor driving avian plumage evolution, with selection favoring either conspicuousness or crypsis (Goméz & Théry, 2007; McNaught & Owens, 2002; Shultz & Burns, 2013). In Steller's Jays, it is possible that brighter and more conspicuous facial features used for visual signaling in open habitats (Rocky Mountain morphotype) may be counterbalanced by selection for darker features (e.g., blue frontal streaks, no superciliary line) that are more cryptic in lower light, closed coastal forest habitats (Coastal morphotype). Plumage traits of individual Coastal Steller's Jays have been found to influence the rate of extra‐pair parentage and the proximity of territories to forest edge (Overeem et al., 2014). While studies have shown that selection in different habitats can be an important driver of phenotypic divergence, the outcome may be clade, environment, or sex‐specific (Cicero et al., 2020; Cornuault et al., 2015; Mason & Bowie, 2020; Medina et al., 2017).

Population density and social behavior also may influence visual signaling in Steller's Jays. In addition to habitat openness, Brown (1963a) noted that conspecific encounters should vary as a function of population density and intraspecific competition. Data from the North American Breeding Bird Survey (2011–2015, https://www.mbr‐pwrc.usgs.gov/bbs/ra2015/ra04780.htm; Sauer et al., 2017) show that the highest density of Steller's Jays occurs in the range of the Coastal morphotype, with notably lower densities in the distribution of both Interior and Rocky Mountain morphotypes. Relative abundance during the breeding season varies widely across the species' range, from a high of 46.4 birds per survey route in the Sierra Nevada to a low of 0.1 bird per route in Wyoming (Walker et al., 2020). Personal field experience (CC) corroborates this regional difference in population density—during the collection of specimens for this study, Coastal Steller's Jays were encountered routinely in higher numbers and in social groups compared with further east where the birds were less common and often solitary or in pairs. Studies in coastal California have shown that Steller's Jays exhibit dominance hierarchies that reflect social rank maintained by frequent interactions (Brown, 1963b), and that socially monogamous pairs have extensively overlapping home ranges with neighboring birds congregating at food resources (Kalinowski et al., 2015). Furthermore, Coastal Steller's Jays cache food items in a social context, whereby birds travel larger distances to cache when other birds are present (Kalinowski et al., 2015). Although comparable data are lacking for Interior and Rocky Mountain populations, one might expect Steller's Jays to encounter other individuals less frequently in areas of low population density, especially if they do not occur in social groups. The interaction between population density, habitat openness, behavioral encounters, and visual signaling traits across morphotypes of Steller's Jays requires further study.

Finally, the role of vocalizations for signaling is a critical component of understanding lineage divergence and ecomorphological associations. Song complexity is significantly associated with habitat openness in New World sparrows, which also show stronger phylogenetic signal in behavioral traits (song structure, vocal duets) compared with morphological traits (Cicero et al., 2020). By contrast, visual and vocal signals reportedly evolve independently, with no habitat association, across the diverse family of tanager species (Mason et al., 2014). Steller's Jays are notoriously loud and have a wide variety of vocalizations and mimetic calls that are used in both territorial defense and predator response (Billings et al., 2017; Brown, 1964; Hope, 1980; Walker et al., 2020). Vocal variation among Steller's Jay populations or morphotypes has not been studied, and thus the association and possible trade‐offs (e.g., Mason et al., 2014) of visual versus vocal traits for signaling aggression remain an open question. Future research that explicitly investigates vocal differences between genetically divergent Steller's Jay populations in the context of morphotype, habitat, and population density begs to be pursued.

### 4.3 Population history

This study presents mtDNA and microsatellite data that shed light on the population history of Steller's Jays in the western United States. Divergence estimates based on mtDNA place the split of the Steller's Jay and Blue Jay in the late Miocene or early Pliocene, approximately 6.36 million years ago (95% highest posterior density = 5.20–7.46 mya). Steller's and Blue jays occupy different bioclimatic regimes in western and eastern North America, respectively, with Blue Jays breeding primarily in more humid hardwood habitats east of the Rocky Mountains including in the Great Plains (with some recent westward expansion, Smith et al., 2020). The estimated timing of divergence of these two sister species coincides with a trend toward progressive drying and cooling in the Great Plains that continued to the Pleistocene (Burke et al., 2018; Frye & Leonard, 1957). The Great Plains is well‐known as an area of divergence and secondary contact between western and eastern lineages of birds and other taxa (Reding et al., 2021; Rising, 1983; Swenson & Howard, 2005). Although these two species of jays are known to hybridize (Walker et al., 2020), such events are probably infrequent (Williams & Wheat, 1971).

Within the Steller's Jay, Coastal/Interior and Rocky Mountain lineages show an exceptionally deep split for avian species (7.8% in mtDNA) that dates to approximately 4.3 mya, which places their divergence in the mid‐Pliocene during a period of warmer conditions (Burke et al., 2018). This split also is reflected in the microsatellite clusters. Interestingly, the distribution of these two lineages is coincident with divergent Coastal and Rocky Mountain varieties of ponderosa pine (Latta & Mitton, 1999; Shinneman et al., 2016; Maguire et al., 2018; https://www.conifers.org/pi/Pinus_ponderosa.php) and Douglas fir (*Pseudotsuga* menziesii, Gugger et al., 2010; https://www.conifers.org/pi/Pseudotsuga_menziesii.php), two conifer species dominant in Steller's Jay habitat. Populations of ponderosa pine within the Coastal lineage show further subspecific differentiation (Callaham, 2013a, 2013b) that matches the respective distributions of Coastal and Interior morphotypes of Steller's Jays. Divergence of Coastal (*P. p*. var. *ponderosa*) and Rocky Mountain (*P. p*. var. *scopulorum*) lineages of ponderosa pine is closely associated with climate and especially winter versus summer precipitation regimes, respectively (Shinneman et al., 2016). Historical separation of ponderosa pine is thought to have occurred during the Wisconsin period of glaciation, with only recent (10,000 years) northward expansion as evidenced by the fossil record (Latta & Mitton, 1999). Divergence of Douglas fir varieties, on the other hand, is consistent with an older Pliocene origin that coincides with the orogeny of the Sierra Nevada and Cascade ranges and subsequent xerification of the Columbia Plateau and Great Basin; further variation likely arose during the Pleistocene as a result of repeated isolation and recontact during glacial–interglacial cycles (Gugger et al., 2010). Both Coastal and Rocky Mountain lineages of Douglas fir and ponderosa pine meet in secondary postglacial contact—Douglas fir in north‐central Washington and southern to eastern British Columbia (Gugger et al., 2010), coincident with the break between Coastal and Interior Steller's Jays; ponderosa pine in west‐central Montana (Latta & Mitton, 1999; Shinneman et al., 2016), near (but north of) the contact between Coastal/Interior and Rocky Mountain lineages of Steller's Jay. The striking biogeographic similarity between both species of conifers and Steller's Jays, combined with evidence for strong ecomorphological associations in Steller's Jays as noted above, suggests a shared history of diversification and niche evolution. Steller's Jays have been shown to track their niche in response to temperature change over historical time periods (Tingley et al., 2009).

Numerous glacial refugia and postglacial expansion routes have been hypothesized to explain phylogeographic patterns and contact zones in North America (e.g., Roberts & Hamaan, 2015; Swenson & Howard, 2005). These include: (1) southern refugia in Mexico and the southern/southeastern United States; (2) coastal refugia in California, the Pacific Northwest (e.g., Haida Gwaii), southeastern Alaska, and the maritime northeast; and (3) northern interior refugia in central Alaska, northern Idaho, the Rocky Mountains, and interior basins (Burg et al., 2005; Gugger et al., 2010; Roberts & Hamaan, 2015; Swenson & Howard, 2005; van Els et al., 2012). For example, van Els et al. (2012) proposed that Canada Jays (*Perisoreus canadensis*) were isolated in different Pleistocene refugia with their preferred conifer species (black spruce *Picea mariana* and white spruce *Picea glauca*) to account for range‐wide phylogeographic variation. Molecular and fossil evidence for Douglas fir suggests two coastal refugia and three to four in the Rocky Mountains (Gugger et al., 2010). Ponderosa pine, which is more arid‐adapted, is hypothesized to have occurred in southern coastal, southern interior highland, and Sierra Madre refugia (Roberts & Hamaan, 2015). Microsatellite data from Steller's Jays in the Pacific Northwest (coastal Washington through British Columbia to southeastern Alaska) revealed rapid divergence and postglacial expansion from at least one coastal refugium (Burg et al., 2005). Our species distribution model for the Last Glacial Maximum supports the scenario of multiple glacial refugia for Steller's Jays including coastal California and the Pacific Northwest, the northern Rocky Mountains, the southwestern and southeastern United States, and several areas in Mexico. Furthermore, ranges east of the Cascade‐Sierra Nevada are projected to have been more fragmented than those for Coastal populations, suggesting a more dynamic history of expansion and contraction in the Interior and Rocky Mountains. Geographic differences in historical demography, population size, and gene flow are reflected in our nuclear genetic diversity and private allele analyses, which suggest that Rocky Mountain Steller's Jays (highest diversity and proportion of populations with private alleles) were historically more isolated in separate refugia. Likewise, the relatively low incidence of private alleles in the Interior supports the dynamic history of those populations. A high number of private alleles in the Pacific Northwest population of Steller's Jay (*C. s. carlottae*) on Haida Gwaii (formerly known as the Queen Charlotte Islands) has been interpreted similarly as evidence for isolation on an island refugium during the Pleistocene (Burg et al., 2005).

### 4.4 Species limits and taxonomic recommendations

Baird (1854:118) recognized Rocky Mountain populations of Steller's Jay as a distinct species in his original taxonomic description of *Cyanocitta macrolopha*. Approximately 20 years later, Coues (1871) discussed his experience with the “Long‐crested Jay” *macrolopha* and commented that this species and the Steller's Jay are “so much alike that they might be considered as one species.” That comment may have been the rationale for lumping the two taxa in the first edition of the checklist to North American Birds, which recognized *frontalis* and *macrolopha* as distinct subspecies (American Ornithologists' Union [AOU], 1886). Although that treatment has stood for over a century, we strongly support re‐elevating Rocky Mountain populations to species status.

Species delimitation requires an integrative taxonomy approach with strong geographic sampling that includes putative contact zones (Cicero et al., 2021). In this study, we combined multiple lines of evidence (morphology, plumage, mtDNA, microsatellites, ecological niche models) from over 1000 specimens and 68 populations of Steller's Jays from the Pacific slope to the Rocky Mountains to understand patterns and processes of diversification in this species. Furthermore, we sampled extensively across potential areas of contact in the Pacific Northwest (Coastal vs. Interior morphotypes) and northern Rocky Mountains (Interior vs. Rocky Mountain morphotypes). Our data unequivocally support the recognition of two species of Steller's Jay representing Coastal/Interior and Rocky Mountain morphotypes under both the Biological Species Concept and Phylogenetic Species Concept (Cicero et al., 2021). In addition to their deep genetic divergence, these populations are morphologically and ecologically distinct with evidence of limited gene flow between them. A comparison of mtDNA sequence divergence between pairs of North American avian species showed that Coastal/Interior and Rocky Mountain Steller's Jays rank among the most divergent, and cline analysis showed a sharp break from southwestern Wyoming and southeastern Idaho to northern Utah. The steepest cline was observed in the microsatellite data over a distance of approximately 130 km (between populations 51 and 53), which matches the average cline width for avian hybrid zones in North America (Slager et al., 2020). Finally, the niche modeling data showed that Coastal/Interior and Rocky Mountain morphotypes occupy different bioclimatic regimes, have a preference for different landcover densities (closed and open, respectively), and experienced separate histories in glacial refugia.

The splitting of species requires consideration of alternate English names, as outlined by the Guidelines for English Bird Names published by the American Ornithological Society's Committee on Classification and Nomenclature of North American Birds (https://americanornithology.org/nacc/guidelines‐for‐english‐bird‐names). Specifically, the guidelines state that for “true phylogenetic daughter species formerly treated as a single parental species, the usual policy is to create a new name for each daughter species. This practice is designed to prevent confusion in the literature as to what taxonomic entity the parental name…references.” Prior to 1957, polytypic species of North American birds had English names assigned to subspecies rather than to the species itself (Strickland, 2017). In the case of the Steller's Jay, two alternate names are well‐established in the literature: Blue‐fronted Jay (AOU, 1886) or Blue‐fronted Steller Jay (Grinnell & Miller, 1944) for the Coastal subspecies *frontalis*, and Long‐crested Jay (Baird, 1854; AOU, 1886) or Long‐crested Steller Jay (Grinnell & Miller, 1944) for the Rocky Mountain subspecies *macrolopha*. These names are descriptive of the blue frontal streaking that distinguishes the Coastal/Interior morphotype, and the long crest typical of the Rocky Mountain morphotype. Although White‐fronted Jay is another descriptive name for Rocky Mountain populations and presents a nice contrast with Blue‐fronted Jay, that name has not been established in the literature. In keeping with the English Name Guidelines reference above, and for simplification of the names, we recommend the scientific and English names Blue‐fronted Jay (*Cyanocitta stelleri*) and Long‐crested Jay (*Cyanocitta macrolopha*) for Coastal/Interior and Rocky Mountain morphotypes, respectively.

In addition to the populations studied here, our mtDNA data revealed that populations from southern Mexico and Guatemala were strongly divergent (uncorrected ND2 sequence divergence of 3.76%) from those to the north in the Rocky Mountains—a value higher than the median (2.15%) for other North American avian species that we compared. Steller's Jays from those southern populations are phenotypically distinct (Brown, 1963a), most notably with a blue rather than black crest. Because our current study did not focus on these southern populations and included only a few samples as outgroups, we cannot recommend further taxonomic action at this time. More detailed study with dense sampling is needed to assess genetic differences, timing of divergence, and potential zones of intergradation between Rocky Mountain, Mexican, and Central American populations. Although these populations are part of the Rocky Mountain clade, we suspect that such a study will provide evidence for additional splitting in future.

## AUTHOR CONTRIBUTIONS

**Carla Cicero:** Conceptualization (lead); data curation (equal); funding acquisition (equal); investigation (equal); methodology (equal); project administration (lead); supervision (lead); visualization (equal); writing – original draft (lead); writing – review and editing (equal). **Nicholas A. Mason:** Data curation (equal); formal analysis (equal); methodology (equal); visualization (equal); writing – review and editing (equal). **Zheng Oong:** Data curation (equal); formal analysis (equal); investigation (equal); methodology (equal); visualization (equal); writing – review and editing (equal). **Pascal O. Title:** Data curation (equal); formal analysis (equal); investigation (equal); methodology (equal); visualization (equal); writing – review and editing (equal). **Melissa E. Morales:** Investigation (equal). **Kevin A. Feldheim:** Investigation (equal); writing – review and editing (equal). **Michelle S. Koo:** Investigation (equal); writing – review and editing (equal). **Rauri C. K. Bowie:** Conceptualization (equal); formal analysis (equal); funding acquisition (equal); investigation (equal); methodology (equal); supervision (equal); writing – review and editing (equal).

## CONFLICT OF INTEREST

The authors declare that they have no conflict of interest.

### OPEN RESEARCH BADGES

This article has earned an Open Data badge for making publicly available the digitally‐shareable data necessary to reproduce the reported results. The data is available at https://doi.org/10.6078/D14Q5N.

## Supporting information


Table S1

Table S2

Table S3

Table S4

Table S5

Table S6

Table S7

## Data Availability

DNA sequences are deposited in GenBank, accessions OM689560–OM690612, OM817568–OM817600 (Table S1). Sampling locations, morphological data, microsatellite genotypes, species distribution modeling occurrence points, and all data files and R code can be accessed in Dryad, https://doi.org/10.6078/D14Q5N. Supplemental Tables [Link], [Link], [Link], [Link] and [Link], [Link] are deposited in Dryad. Supplemental Table S5 is deposited in Zenodo (https://doi.org/10.5281/zenodo.7311504).

## APPENDIX 1

**TABLE A1:** Population samples and corresponding ND2 lineage for cline analysis of Steller's Jays.

Population number	Population name	Distance from ID_Bonner (km)	ND2 lineage	
			Coastal (N)	Rocky Mtn. (N)
41	ID_Bonner	0	10	0
43	MT_Missoula	221.3	8	0
49	ID_Lemhi	358.7	5	0
50	WY_Teton	670.1	10	2
51	ID_BearLake	759	5	5
52	UT_Cache	803.4	4	2
53	UT_Weber	864.3	3	6
54	UT_Wasatch	950.8	0	10
55	UT_Utah	1003	2	12
60	UT_Sanpete	1073	0	15
61	UT_BoulderMtn	1201	0	15

*Note*: Population numbers correspond to those in Figure 1 and Table S1. ND2 lineage shows the number of individuals with either the Coastal (including Interior) or Rocky Mountain haplotype in that population.

**TABLE A2:** Species pairs, Genbank accessions, and associated specimen collection numbers (if known) used in pairwise comparisons of ND2 sequence divergence.

**Species 1a: *Anser caerulescens***	**Species 1b: *Anser rossii***
FJ423761.1	EU585683.1
**Species 2a: *Colinus cristatus***	**Species 2b: *Colinus virginianus***
KC556536.1—LACM 40829	KC556552.1—AMNH 783835
KC556535.1—FMNH 425599	KC556551.1—MMNH 12383
KC556534.1—LACM 36146	KC556550.1—YPM 21703
KC556531.1—LACM 35923	KC556549.1—UCM 1148
KC556529.1—FMNH 400209	KC556548.1—UCLA 31251
KC556528.1—FMNH 103886	KC556547.1—AMNH 778227
KC556527.1—AMNH 186633	KC556546.1—MMNH 12384
KC556526.1—LACM 36149	KC556545.1—YPM 21193
KC556525.1—FMNH 103887	KC556544.1—LACM 21226
KC555969.1—FMNH 400045	KC556539.1—AMNH 776246
	KC556538.1—AMNH 775890
	KC556537.1—UCLA 23470
	KC556211.1
	KC556204.1
	AF028776.1—MMNH 51080
**Species 3a: *Lagopus mutus***	**Species 3b: *Lagopus lagopus***
AF230118.1	AF230116.1
**Species 4a: *Centrocercus urophasianus***	**Species 4b: *Centrocercus minimus***
MW574357.1—UF 47008 AF230123.1	KU094635.1—USNM 601339
**Species 5a: *Rallus longirostris***	**Species 5b: *Rallus elegans***
KM026530.1 KJ481905.1 KJ481904.1 KJ481903.1 KJ481902.1 KJ481901.1 KP081624.1—UMMZ 620 KP081623.1—LSUMZ 182869 KP081622.1—LSUMZ 182868 KP081621.1—LSUMZ 205068 KP081620.1—LSUMZ 182867 KP081619.1—LSUMZ 204226 KP081618.1—LSUMZ 204224	MG981829.1 MG981828.1 MG981823.1 MG981788.1 MG981782.1 MG981778.1 MG981763.1 KP081617.1—LSUMZ B–71309 KP081616.1—LSUMZ B–71308 KP081615.1—LSUMZ B–71305 KP081590.1—LSUMZ 184825 KP081589.1—LSUMZ 184824 KP081588.1—LSUMZ 184823 KP081587.1—LSUMZ 184822 KP081586.1—LSUMZ 184821 KP081585.1—LSUMZ 184820 KP081584.1—LSUMZ 184819 KP081583.1—LSUMZ 184818 KP081582.1—LSUMZ 184826 KP081581.1—LSUMZ 184817
**Species 6a: *Limnodromus griseus***	**Species 6b: *Limnodromus scolopaceus***
JQ963026.1	EF373248.1—ROM 157030
**Species 7a: *Megascops kennicottii***	**Species 7b: *Megascops asio***
KT799359.1—ANSP 192422 JF909892.1—CAS 90394 EU601053.1—MVZ 182896	KT799334.1—FMNH 440242 EU601054.1—MVZ 179828
**Species 8a: *Sphyrapicus ruber***	**Species 8b: *Sphyrapicus nuchalis***
DQ361285.1—USNM 621107 KT455645.1—MMNH 47193	MF766713.1—LSU B–55515 FJ161065.1– MZFC 13194 DQ479166.1—KU 91177
**Species 9a: *Contopus sordidulus***	**Species 9b: *Contopus virens***
MG722594.1—ZMUC B–127969 MG722593.1—UWBM 61800 AF447635.1—MVZ 168549 AF447634.1—MVZ 169024	MG722591.1—UWBM 74094 AF447637.1—MVZ 168655 AF447636.1—MVZ 168652
**Species 10a: *Empidonax alnorum***	**Species 10b: *Empidonax traillii***
MG722574.1—ZMUC B–125779 AY143215.1—MVZ 177237 AY143214.1—MVZ 168682 AY143213.1—MVZ 168678	MG722575.1—UWBM 73093 AY143230.1—MVZ 168696 AY143229.1—MVZ 168697
**Species 11a: *Vireo solitarius***	**Species 11b: *Vireo cassinii***
AY030137.1—MVZ 168741 KM115394.1—UWBM 105473 KM115393.1—MMNH 42753 KM115392.1—UWBM 105473	KM115293.1—UWBM 115620 KM115292.1—UWBM 110859 KM115291.1—UWBM 106197 KM115290.1—UWBM 110722
**Species 12a: *Aphelocoma woodhouseii***	**Species 12b: *Aphelocoma californica***
HQ123779.1—FMNH 343488	HQ123766.1—FMNH 333909
HQ123778.1—FMNH 343478	HQ123765.1—FMNH 333904
HQ123777.1—FMNH 343467	HQ123764.1—FMNH 333880
HQ123776.1—FMNH 343452	HQ123763.1—FMNH 333883
HQ123775.1—FMNH 334229	HQ123760.1—FMNH 342048
HQ123774.1—FMNH 334226	HQ123759.1—FMNH 343428
HQ123773.1—FMNH 334157	HQ123758.1—FMNH 333845
HQ123772.1—FMNH 334212	HQ123757.1—FMNH 333867
HQ123771.1—FMNH 334181	HQ123756.1—FMNH 334005
HQ123770.1—FMNH 334109	HQ123755.1—FMNH 343436
HQ123768.1—FMNH 334083	HQ123754.1—FMNH 343443
AY030142.1—MVZ 166923	HQ123753.1—FMNH 343440
**Species 13a: *Pica nuttalli***	**Species 13b: *Pica hudsonia***
MG641021.1—ZMUC 68942 MG641020.1—AMNH SKIN 836027 MG641019.1—AMNH SKEL 26711 MG641018.1—AMNH SKEL 26725 MG641017.1—AMNH SKEL 26710	MG641031.1—ZMUC 68939 MG641030.1—ZMUC 68938 MG641029.1—ZMUC 68937 MG641028.1—AMNH DOT10124 MG641027.1—AMNH SKEL 25322 MG641026.1—UWBM 72363 MG641025.1—UWBM 78117 MG641024.1—UWBM 78146 MG641023.1—UWBM 80453 MG641022.1—UWBM 70873 HM776970.1—UAM 13238 HM640851.1—UAM 13049 HM640850.1—UAM 10142 HM640849.1—UAM 10140 HM640848.1—UAM 14665 HM640847.1—UAM 13053 HM640846.1—UAM 12453 HM640845.1—UAM 13052 HM640844.1—UAM 10141 HM640843.1—UAM 10139
**Species 14a: *Baeolophus inornatus***	**Species 14b: *Baeolophus ridgwayi***
KF183886.1—MVZ 173182 KF183885.1—MVZ 172903	KF183887.1—MVZ 171985
**Species 15a: *Baeolophus bicolor***	**Species 15b: *Baeolophus atricristatus***
AY825995.1—ANSP 182213 KF183884.1—MVZ 178245 KF183883.1—NRM 20086098	KF183882.1—NRM 570162
**Species 16a: *Polioptila californica***	**Species 16b: *Polioptila melanura***
MG903081.1—AMNH SKEL 24399 MG903080.1 MG903079.1—AMNH 132165 AF027850.1 AF027849.1—MMNH 50732 KC864535.1 KC864534.1 KC864533.1—AMNH SKEL 24470 KC864532.1 KC864531.1 KC864530.1—MMNH 50732 KC864529.1—MMNH 50735 KC864528.1—AMNH SKEL 24402 KC864527.1 KC864526.1 KC864525.1 KC864524.1—AMNH SKEL 24406 KC864523.1—AMNH SKEL 24466 KC864522.1	MG903132.1 MG903131.1—LSUMZ 130678 MG903130.1—UWBM 114779 MG903129.1—UWBM 108513 MG903128.1—UWBM 108511 MG903127.1—UWBM 108509 MG903126.1—UWBM 108508 MG903125.1—UWBM 108507 MG903124.1—UWBM 112001 MG903123.1—UWBM 111999 MG903122.1—UWBM 111996 MG903121.1—UWBM 110568 MG903120.1—UWBM 110567 MG903119.1—UWBM 106658 MG903118.1—LSUMZ 217010 MG903117.1—MMNH 45791 MG903116.1—MMNH 45790 MG903115.1 MG903114.1 AF027859.1—LSUMZ 156098
**Species 17a: *Toxostoma cinereum***	**Species 17b: *Toxostoma bendirei***
EF468208.1	JN799681.1 JN799680.1 AF287161.1
**Species 18a: *Catharus minimus***	**Species 18b: *Catharus bicknelli***
KY995108.1—NYSM zt– 1480 MG966819.1 MG966818.1 MG966817.1 MG966816.1 MG966815.1 MG966814.1 MG966813.1 MG966812.1 MG966811.1 MG966810.1 MG966809.1 MG966808.1 MG966807.1 MG966806.1 MG966805.1 MG966804.1 MG966803.1 MG966802.1 MG966801.1	KY995052.1—NYSM zo–15,345 KY995051.1—NYSM zo–14,314 KY995050.1—NYSM zo–13,331 KY995049.1—NYSM zo–11,235 KY995048.1—NYSM zt–1498 KY995047.1—NYSM zt–1497 KY995046.1—NYSM zt–1496 KY995045.1—NYSM zt–1495 KY995044.1—NYSM zt–1494 KY995043.1—NYSM zt–1493 KY995042.1—NYSM zt–1492 KY995041.1—NYSM zt–1488 KY995040.1—NYSM zt–1487 KY995039.1—NYSM zt–1486 KY995038.1—NYSM zt–1485 KY995037.1—NYSM zt–1484 KY995036.1—NYSM zt–1483 KY995035.1—NYSM zt–1407 KY995034.1—NYSM zt–1478 KY995033.1—NYSM zt–1475
**Species 19a: *Acanthis flammea***	**Species 19b: *Acanthis hornemanni***
FJ547507.1—USNM 640217 NC_027285.1 KR422696.1 JN715417.1—NRM 20016449	JN715419.1—AJN 000043
**Species 20a: Ammodramus caudacutus**	**Species 20b: Ammodramus nelsoni**
DQ459539.1	DQ459542.1—MMNH 43267
**Species 21a: *Junco hyemalis***	**Species 21b: *Junco phaeonotus***
FJ236293.1 KX461780.1 KX461779.1 KX461778.1 KX461777.1 KX461776.1 KX461775.1 KX461720.1 KX461719.1 KX461718.1 KX461717.1 KX461716.1 KX461715.1 KX461714.1 KX461713.1 KX461712.1 KX461711.1 KX461710.1 KX461709.1 KX461708.1	MF458395.1—FMNH 394076 KX461774.1 KX461773.1 KX461772.1 KX461771.1 KX461770.1 KX461769.1 KX461768.1 KX461767.1 KX461766.1 KX461765.1 KX461764.1 KX461763.1 KX461762.1 KX461761.1 KX461760.1 KX461759.1 KX461758.1 KX461734.1 KX461733.1
**Species 22a: *Zonotrichia leucophrys***	**Species 22b*: Zonotrichia atricapilla***
FJ236292.1—MVZ 169413 GQ205561.1 GQ205560.1 GQ205559.1 GQ205558.1 GQ205557.1 GQ205556.1 GQ205555.1 GQ205554.1 GQ205553.1 GQ205552.1 GQ205551.1 GQ205550.1 GQ205549.1 GQ205548.1 GQ205547.1 GQ205546.1 GQ205545.1 GQ205544.1 GQ205543.1	MT050828.1—UWBM 116866 GQ205507.1 GQ205506.1 GQ205505.1—UWBM 83465 GQ205504.1—UWBM 83464 GQ205503.1—UWBM 81808 GQ205502.1—UWBM 81767 GQ205501.1—UWBM 80465 GQ205500.1—UWBM 80425 GQ205499.1—UWBM 80383 GQ205498.1—UWBM 80072 GQ205497.1—UWBM 79613 GQ205496.1—UWBM 79560 GQ205495.1—UWBM 79432 GQ205494.1—UWBM 79419 GQ205493.1—UWBM 79416 GQ205492.1—UWBM 79307 GQ205491.1—UWBM 79260 GQ205484.1—UWBM 70567 GQ205483.1—UWBM 70566
**Species 23a: *Artemisiospiza belli***	**Species 23b: *Artemisiospiza nevadensis***
MH460923.1—SDNHM 50647 MH460922.1—MVZ 35748 MH460921.1—MVZ 35744 MH460920.1—MVZ 35743 MH460919.1—MVZ 170308 MH460918.1—MVZ 170307 MH460917.1—MVZ 169356 MH460916.1—MVZ 169355 MH460915.1—MVZ 170285 MH460914.1—MVZ 170335 MH460913.1—MVZ 170334 MH460912.1—MVZ 169093 MH460911.1—MVZ 169092 MH460910.1—MVZ 170009 MH460909.1—MVZ 170008 MH460908.1—MVZ 170611 MH460907.1—MVZ 170610 MH460906.1—MVZ 170595 MH460905.1—MVZ 170594 MH460904.1—MVZ 169980	MH460932.1—MVZ 166949 MH460931.1—MVZ 166948 MH460930.1—MVZ 170234 MH460929.1—MVZ 170233 MH460928.1—MVZ 167151 MH460927.1—MVZ 170340 MH460926.1—MVZ 170339 MH460925.1—MVZ 166961 MH460924.1—MVZ 166960
**Species 24a: *Pipilo maculatus***	**Species 24b: *Pipilo erythrophthalmus***
MN593806.1—MVZ 177601 FJ236291.1 FJ547300.1—UWBM 98298	FJ547301.1—UWBM 120436
**Species 25a: *Sturnella magna*** **(split by the American Ornithological Society in 2022; some sequences may represent *Sturnella lilianae*)**	**Species 25b: *Sturnella neglecta***
FJ154704.1—MSB 22889 FJ154703.1—MSB 19558 FJ154696.1—FMNH 393588 FJ154695.1—FMNH 387734 FJ154694.1—FMNH 339779 FJ154693.1—FMNH 356947 FJ154692.1—STRIBC 5004 FJ154691.1—FMNH 393903 FJ154690.1—LSUMZ 177781 FJ154689.1—CUMV 50729 FJ154688.1—MMNH 45900 FJ154687.1—MSB 24299 FJ154686.1—MSB 23221 FJ154685.1—MSB 23222 FJ154684.1—MSB 22311 FJ154683.1—MSB 21208 FJ154682.1—MSB 21206 FJ154681.1—MSB 21210 FJ154680.1—FMNH 350659 FJ154679.1—MSB 22221	MT050825.1—UWBM 90472 FJ236288.1—MVZ 177228 FJ154705.1—AMNH SKEL 26268 FJ154702.1—MSB 20381 FJ154701.1—MMNH 43739 FJ154700.1—MMNH 43737 FJ154699.1—MMNH 43736 FJ154698.1—FMNH 341967 FJ154697.1—FMNH 341966 FJ154677.1—MSB 21226 AF290127.1
**Species 26a: *Quiscalus major***	**Species 26b: *Quiscalus mexicanus***
EU414609.1—UWBM 108556 EU414608.1—UWBM 108591 EU414607.1—UWBM 108569 EU414606.1—UWBM 108568 EU414605.1—UWBM 108567 EU414604.1—UWBM 108565 EU414603.1—UWBM 108543 EU414602.1—UWBM 108541 EU414601.1—UWBM 108539 EU414600.1—UWBM 108538 EU414599.1—UWBM 108576 EU414598.1—UWBM 108572 EU414597.1—UWBM 108563 EU414596.1—UWBM 108559 AF109953.1	NC_051021.1 MN356197.1 EU414595.1 EU414594.1—UWBM 108118 EU414593.1—UWBM 108111 EU414592.1—UWBM 108110 EU414591.1—UWBM 70113 EU414590.1—UWBM 70237 EU414589.1—UWBM 70227 EU414588.1—UWBM 103496 EU414587.1—UWBM 103459 EU414586.1—UWBM 103428 EU414585.1—WFVZ 54953 EU414584.1—WFVZ 54954 EU414583.1—WFVZ 54952 EU414582.1—WFVZ 54955 EU414581.1 EU414580.1—UWBM 105440 EU414579.1—UWBM 108707 EU414578.1—UWBM 108706
**Species 27a: *Setophaga townsendi***	**Species 27b: *Setophaga occidentalis***
GU932101.1—UWBM 41918 FJ374120.1—UWBM 84873 FJ374119.1—UWBM 84874 FJ374118.1—UWBM 84875 FJ374117.1—UWBM 84876 FJ374116.1—UWBM 84877 FJ374115.1—UWBM 84878 FJ374114.1—UWBM 84879 FJ374113.1—UWBM 84885 FJ374112.1—UWBM 84886 FJ374111.1—UWBM 84887 FJ374110.1—UWBM 84888 FJ374109.1—UWBM 84889 FJ374108.1—UWBM 84890 FJ374107.1—UWBM 84891 FJ374106.1—UWBM 84892 FJ374105.1 FJ374104.1	EU815770.1 GU932091.1—UWBM 46693 FJ373895.1—UWBM 66140
**Species 28a: *Piranga ludoviciana***	**Species 28b: *Piranga bidentata***
FJ236296.1 AF290109.1	MH700646.1
**Species 29a: *Passerina caerulea***	**Species 29b: *Passerina amoena***
EF529882.1—LSUMZ 154263	MN356416.1 EF529886.1—MMNH 45514 FJ236297.1
**Species 30a: *Passerina versicolor***	**Species 30b: *Passerina ciris***
EF529888.1—UWBM 57374	EF529883.1—LSUMZ 117711

*Note*: Voucher numbers are given when possible, and only specimens or samples known to be deposited in museum collections are referenced for citation purposes. Institution codes are: AJN, Ajtte Swedish Mountain and Sami Museum; AMNH, American Museum of Natural History; CAS, California Academy of Sciences; CUMV, Cornell University Museum of Vertebrates; FMNH, Field Museum of Natural History; LACM, Natural History Museum of Los Angeles County; LSUMZ, Louisiana State University Museum of Natural Science; MMNH, Bell Museum of Natural History; MSB, Museum of Southwestern Biology; MVZ, Museum of Vertebrate Zoology; MZFC, Museo de Zoología Alfonso L. Herrera, Facultad de Ciencias, Universidad Nacional Autónoma de México; NRM, Swedish Museum of Natural History; NYSM, New York State Museum; ROM, Royal Ontario Museum; STRIBC, Smithsonian Tropical Research Bird Collection; UAM, University of Alaska Museum of the North; UCLA, University of California Los Angeles; UCM, University of Colorado Museum of Natural History; UF, University of Florida Museum of Natural History; UMMZ, University of Michigan Museum of Zoology; USNM, United States National Museum; UWBM, University of Washington Burke Museum; YPM, Yale University Peabody Museum; ZMUC, University of Copenhagen Zoological Museum.

**TABLE A3:** Full list of environmental variables used in species distribution modeling of Steller's Jays

Variable	Source
bioclim 1: annual mean temperature	CHELSA v1.2; Karger et al. (2017)
bioclim 2: mean diurnal range	CHELSA v1.2; Karger et al. (2017)
bioclim 3: isothermality	CHELSA v1.2; Karger et al. (2017)
bioclim 4: temperature seasonality	CHELSA v1.2; Karger et al. (2017)
bioclim 5: max temp. of warmest month	CHELSA v1.2; Karger et al. (2017)
bioclim 6: min temp. of coldest month	CHELSA v1.2; Karger et al. (2017)
bioclim 7: temperature annual range	CHELSA v1.2; Karger et al. (2017)
bioclim 8: mean temp. of wettest quarter	CHELSA v1.2; Karger et al. (2017)
bioclim 9: mean temp. pf driest quarter	CHELSA v1.2; Karger et al. (2017)
bioclim 10: mean temp. of warmest quarter	CHELSA v1.2; Karger et al. (2017)
bioclim 11: mean temp. of coldest quarter	CHELSA v1.2; Karger et al. (2017)
bioclim 12: annual precipitation	CHELSA v1.2; Karger et al. (2017)
bioclim 13: precip. of wettest month	CHELSA v1.2; Karger et al. (2017)
bioclim 14: precip. of driest month	CHELSA v1.2; Karger et al. (2017)
bioclim 15: precip. Seasonality	CHELSA v1.2; Karger et al. (2017)
bioclim 16: precip. of wettest quarter	CHELSA v1.2; Karger et al. (2017)
bioclim 17: precip. of driest quarter	CHELSA v1.2; Karger et al. (2017)
bioclim 18: precip. of warmest quarter	CHELSA v1.2; Karger et al. (2017)
bioclim 19: precip. of coldest quarter	CHELSA v1.2; Karger et al. (2017)
annual PET	ENVIREM; Title and Bemmels (2018)
climatic moisture index	ENVIREM; Title and Bemmels (2018)
continentality	ENVIREM; Title and Bemmels (2018)
emberger's Q	ENVIREM; Title and Bemmels (2018)
growing degree days above 0C	ENVIREM; Title and Bemmels (2018)
growing degree days above 5C	ENVIREM; Title and Bemmels (2018)
max temp. of coldest month	ENVIREM; Title and Bemmels (2018)
min temp. of warmest month	ENVIREM; Title and Bemmels (2018)
PET of coldest quarter	ENVIREM; Title and Bemmels (2018)
PET of driest quarter	ENVIREM; Title and Bemmels (2018)
PET seasonality	ENVIREM; Title and Bemmels (2018)
PET of warmest quarter	ENVIREM; Title and Bemmels (2018)
PET of wettest quarter	ENVIREM; Title and Bemmels (2018)
thermicity index	ENVIREM; Title and Bemmels (2018)
landcover 1: evergreen/deciduous needleleaf trees	Tuanmu and Jetz (2014)
landcover 2: evergreen broadleaf trees	Tuanmu and Jetz (2014)
landcover 3: deciduous broadleaf trees	Tuanmu and Jetz (2014)
landcover 4: mixed/other trees	Tuanmu and Jetz (2014)
landcover 5: shrubs	Tuanmu and Jetz (2014)
landcover 6: herbaceous vegetation	Tuanmu and Jetz (2014)
landcover 8: regularly flooded vegetation	Tuanmu and Jetz (2014)
landcover 10: snow/ice	Tuanmu and Jetz (2014)
landcover 11: barren	Tuanmu and Jetz (2014)

**TABLE A4:** Loadings on the first two principal components of morphological variation in Steller's Jays.

Morphological character	PC1 (43.42%)	PC2 (23.98%)
Wing length	0.334	0.436
Tail length	0.533	0.581
Tarsus plus toe length	0.146	−0.077
Bill length	0.042	−0.025
Bill depth	0.025	−0.024
Bill width	0.010	−0.029
Crest length	0.100	0.278
Body mass	0.755	−0.622

*Note*: The percentage of variation explained by each PC axis is given in parentheses.

**TABLE A5:** Results from the linear mixed models (LMM) of variation in morphologic traits.

Effect	Value	*T* value	*p*‐Value	$R_{m}^{2}$	$R_{c}^{2}$
*Wing length (mm)*					
(Intercept)	136.81 ± 0.48	283.08	**<.001**	.53	.64
Sex (Male)	5.07 ± 0.23	21.97	**<.001**		
Age (Adult)	2.24 ± 0.35	6.42	**<.001**		
Morphotype (Interior)	7.84 ± 0.75	10.49	**<.001**		
Morphotype (Rocky Mtn.)	5.08 ± 0.71	7.18	**<.001**		
*Tail length (mm)*					
(Intercept)	136.14 ± 0.73	186.77	**<.001**	.54	.71
Sex (Male)	5.45 ± 0.30	17.96	**<.001**		
Age (Adult)	3.83 ± 0.46	8.30	**<.001**		
Morphotype (Interior)	13.84 ± 1.23	11.23	**<.001**		
Morphotype (Rocky Mtn.)	1.98 ± 1.17	1.70	.1		
*Bill length (mm)*					
(Intercept)	20.19 ± 0.14	144.83	**<.001**	.19	.41
Sex (Male)	0.99 ± 0.06	15.39	**<.001**		
Age (Adult)	0.44 ± 0.10	4.42	**<.001**		
Morphotype (Interior)	−0.31 ± 0.22	−1.40	.17		
Morphotype (Rocky Mtn.)	0.1 ± 0.21	0.48	.63		
*Bill depth (mm)*					
(Intercept)	8.71 ± 0.06	136.51	**<.001**	.23	.62
Sex (Male)	0.42 ± 0.02	18.66	**<.001**		
Age (Adult)	0.17 ± 0.03	5.07	**<.001**		
Morphotype (Interior)	−0.13 ± 0.12	−1.11	.27		
Morphotype (Rocky Mtn.)	−0.34 ± 0.11	−3.13	**<.001**		
*Bill width (mm)*					
(Intercept)	8.95 ± 0.05	179.95	**<.001**	.33	.45
Sex (Male)	0.31 ± 0.03	11.84	**<.001**		
Age (Adult)	0.11 ± 0.04	2.94	**<.001**		
Morphotype (Interior)	−0.44 ± 0.07	−6.13	**<.001**		
Morphotype (Rocky Mtn.)	−0.58 ± 0.07	−8.50	**<.001**		
*Crest length (mm)*					
(Intercept)	39.94 ± 0.38	103.99	**<.001**	.43	.56
Sex (Male)	1.82 ± 0.18	9.97	**<.001**		
Age (Adult)	1.52 ± 0.30	5.13	**<.001**		
Morphotype (Interior)	2.83 ± 0.54	5.21	**<.001**		
Morphotype (Rocky Mtn.)	6.37 ± 0.52	12.25	**<.001**		
*Body mass (g)*					
(Intercept)	110.96 ± 1.33	83.55	**<.001**	.26	.70
Sex (Male)	8.52 ± 0.40	21.06	**<.001**		
Age (Adult)	−0.2 ± 0.63	−0.32	.75		
Morphotype (Interior)	5.49 ± 2.51	2.19	**.03**		
Morphotype (Rocky Mtn.)	−6.58 ± 2.36	−2.79	**.01**		
*PC2*					
Intercept	−6.38 ± 0.79	−8.12	**<.001**	.37	.54
Sex (Male)	0.53 ± 0.36	1.47	.14		
Age (Adult)	3.36 ± 0.59	5.65	**<.001**		
Morphotype (Interior)	8.84 ± 1.13	7.83	**<.001**		
Morphotype (Rocky Mtn.)	9.38 ± 1.10	8.55	**<.001**		

*Note*: In each model, population was included as a random grouping effect because multiple individuals were sampled from the same population or locality. *p*‐Values that fall below the .05 threshold are considered statistically significant and shown in bold. The effects of each factor are compared with a base model that describes variation among female, juvenile birds from Coastal morphotype populations. The marginal ($R_{m}^{2}$) and conditional ($R_{c}^{2}$) coefficients of the models also are shown.

**TABLE A6:** Variability in 12 microsatellite loci across three Steller's Jay morphotypes.

Locus	*n*	*A*	*R*_S_	*H*_O_	*H*_E_	*p*
*Coastal*						
Cst7	682	20	17.591	0.87537	0.90984	.04681
Cst12	678	8	7.553	0.76696	0.79531	.06940
Cst16	681	8	7.558	0.72247	0.74286	.91013
Cst24	681	11	10.032	0.78267	0.80911	.15883
Cst45	681	17	14.458	0.86197	0.86443	.11892
Cst46	681	10	9.088	0.77974	0.78078	.33781
Cst58	682	16	11.762	0.79619	0.81734	.20601
Cst61	681	12	10.416	0.79442	0.79026	.04961
Cst87	681	11	9.108	0.79442	0.76377	.54995
Cst103	679	16	13.324	0.83652	0.85117	.01564
Cst117	682	16	14.242	0.88710	0.88632	.33805
Cst120	682	8	7.780	0.79179	0.79385	.34307
*Interior*						
Cst7	166	22	21.988	0.92169	0.92769	.92597
Cst12	166	8	7.988	0.75301	0.78073	.41624
Cst16	166	8	7.988	0.71084	0.72981	.42763
Cst24	166	12	11.952	0.81325	0.81505	.19474
Cst45	166	13	12.988	0.81325	0.78459	.71176
Cst46	166	9	8.976	0.77711	0.77835	.47276
Cst58	166	10	9.988	0.80120	0.82506	.28086
Cst61	166	10	9.988	0.84337	0.81189	.94284
Cst87	166	10	9.976	0.75301	0.78923	.11818
Cst103	166	14	13.988	0.92169	0.90201	.12127
Cst117	166	16	15.976	0.87349	0.88230	.42431
Cst120	166	8	7.976	0.74699	0.79316	.10856
*Rocky Mountain*						
Cst7	165	17	16.994	0.84848	0.91112	.34482
Cst12	165	10	9.988	0.78182	0.79523	.34358
Cst16	165	10	10.000	0.72121	0.72046	.88104
Cst24	165	15	14.994	0.86061	0.85851	.97403
Cst45	164	13	13.000	0.71951	0.75806	.02989
Cst46	165	11	10.994	0.78182	0.81336	.71257
Cst58	165	12	11.994	0.73333	0.85779	.00282*
Cst61	165	9	8.988	0.80606	0.77952	.80341
Cst87	165	11	10.994	0.84242	0.84440	.52608
Cst103	165	12	11.976	0.81818	0.79149	.71894
Cst117	165	14	13.994	0.87273	0.87929	.79006
Cst120	165	10	9.988	0.76970	0.77550	.07814

*Note*: Included are the number of individuals genotyped for each locus (*n*), total number of alleles at each locus (A), allelic richness estimates (*R*_S_), observed heterozygosity (*H*_O_), expected heterozygosity (*H*_E_), and associated *p‐*values. Asterisk indicates significant deviation between *H*_O_ and *H*_E_ after the application of Holm–Bonferroni sequential corrections (*p* < .004).

**TABLE A7:** Likelihood and Delta *K* (Δ*K*) values for different numbers of clusters (*K*) based on 12 microsatellite loci.

*K*	Mean Ln*P*(*K*)	Stdev Ln*P*(*K*)	Ln′(*K*)	|Ln″(*K*)|	Δ*K*
*All populations*					
1	−51571.10	0	NA	NA	NA
2	−50431.47	3.8373	1139.63	822.89	**214.44735**
3	−50114.73	13.6712	316.74	286.73	20.97325
4	**−50084.72**	48.6786	30.01	115.02	2.36285
5	−50169.73	141.0371	−85.01	42.14	0.29879
6	−50212.6	186.485	−42.87	60.12	0.32239
7	−50315.59	238.0077	−102.99	211.95	0.89052
8	−50630.53	347.1052	−314.94	186.22	0.53649
9	−50759.25	771.1563	−128.72	198.11	0.25690
10	−50689.86	274.7666	69.39	511.41	1.86125
11	−51131.88	382.9676	−442.02	688.54	1.79791
12	−50885.36	353.7627	246.52	637.14	1.80104
13	−51275.98	463.0973	−390.62	135.12	0.29178
14	−51531.48	704.1627	−255.5	306.66	0.43550
15	−51480.32	555.7407	51.16	901.22	1.62166
16	−52330.38	1174.5225	−850.06	849.50	0.72327
17	−52330.94	785.5853	−0.56	349.14	0.44443
18	−52680.64	1280.6428	−349.70	1135.74	0.88685
19	−51894.6	800.3555	786.04	1880.66	2.34978
20	−52989.22	1473.5755	1094.62	891.28	0.60484
21	−54975.12	1408.9451	−1985.90	2504.98	1.77791
22	−54456.04	2213.1076	519.08	662.62	0.29941
23	−54599.58	4879.5397	−143.54	1182.30	0.24230
24	−53560.82	2238.8534	1038.76	1443.92	0.64494
25	−53965.98	1804.415	−405.16	NA	NA
*Coastal only*					
1	−31442.20	0	NA	NA	NA
2	−31398.43	8.3955	43.77	10.23	1.21851
3	**−31344.43**	35.767	54.00	122.44	**3.42327**
4	−31412.87	48.8935	−68.44	37.22	0.76125
5	−31518.53	221.5186	105.66	145.55	0.65707
6	−31478.64	77.6566	39.89	206.71	2.66185
7	−31645.46	187.0956	166.82	136.00	0.72690
8	−31676.28	281.2666	−30.82	213.25	0.75818
9	−31920.35	407.1814	244.07	419.34	1.02986
10	−32583.76	575.8249	663.41	NA	NA
*Interior only*					
1	**−7795.94**	0.3864	NA	NA	NA
2	−7888.17‐	37.7755	−92.23	21.25	0.56253
3	−8001.65	69.9344	−113.48	141.42	**2.02218**
4	−7973.71	87.3833	27.94	125.41	1.43517
5	−8071.18	73.8661	−97.47	123.64	1.67384
6	−8045.01	67.0508	26.17	47.44	0.70752
7	−8066.28	131.6808	−21.27	117.45	0.89193
8	−7970.10	69.7415	96.18	88.15	1.26395
9	−7962.07	90.595	8.03	11.72	0.12937
10	−7965.76	117.2533	−3.69	NA	NA
*Coastal plus Interior*					
1	−40183.30	0	NA	NA	NA
2	−39883.56	5.0271	299.74	245.41	**48.81758**
3	**−39829.23**	15.7564	54.33	96.74	6.13972
4	−39871.64	91.5779	−42.41	8.25	0.09009
5	−39905.80	151.9963	−34.16	64.15	0.42205
6	−40004.11	152.4607	−98.31	87.45	0.57359
7	−40014.97	216.1109	−10.86	154.83	0.71644
8	−40180.66	273.7331	165.69	62.77	0.22931
9	−40409.12	347.931	228.46	73.63	0.21162
10	−40711.21	640.3579	302.09	NA	NA
*Rocky Mountain only*					
1	−7730.61	0.4285	NA	NA	NA
2	**−7722.53**	9.0815	8.08	25.18	**2.77280**
3	−7739.63	52.6531	−17.10	43.21	0.82065
4	−7799.94	41.0054	−60.31	13.17	0.32118
5	−7847.08	64.0229	−47.14	3.22	0.05030
6	−7897.44	38.4136	−50.36	39.11	1.01813
7	−7986.91	95.6359	−89.47	128.41	1.34270
8	−7947.97	61.4705	38.94	47.22	0.76817
9	−7956.25	66.1736	−8.28	13.06	0.19736
10	−7951.47	115.0649	4.78	NA	NA

*Note*: Separate analyses were run for all populations combined and for different combinations based on morphotype (Coastal only, Interior only, Coastal plus Interior, Rocky Mountain only). Values in bold indicate the optimal *K* value for that analysis.

**TABLE A8:** Different HZAR models and associated values for the four traits (microsatellites, ND2 haplotype frequencies, morphology PC1, and blue frontal streak frequencies) that characterize populations of Steller's Jay across geographically coincident clines (Figure 8)

Model	*N*	Center (km)	Width (km)	ΔAICc	Tails	*p*max/*p*min
*Microsatellites*						
Model III	115	940.17	147.73	0	Free	None
Model IX	115			4.4684	Free	Left
Model VII	115			4.5993	Free	Right
Model II	115			9.1183	Fixed	None
Model XV	115			10.3301	Free	Both
Model VIII	115			12.0412	Fixed	Left
Model V	115			13.4523	Fixed	Right
Model XI	115			13.4545	Fixed	Mirror
Model XII	115			14.0971	Free	Mirror
Model XIV	115			16.4456	Fixed	Both
Model XIII	115			19.0088	None	Both
Model VI	115			38.7901	None	Left
Model X	115			54.4056	None	Mirror
Model IV	115			94.8533	None	Right
Model I	115			111.1590	None	None
*ND2 haplotype frequencies*						
Model I	115	869.32	389.06	0	None	None
Model IV	115			4.2565	None	Right
Model VII	115			4.2565	None	Left
Model X	115			4.2565	None	Mirror
Model III	115			4.3126	Free	None
Model XIII	115			8.6706	None	Both
Model XII	115			8.7497	Free	Mirror
Model IX	115			8.7563	Free	Left
Model VI	115			8.7976	Free	Right
Model XV	115			13.2988	Free	Both
*Morphology PC1*						
Model II	83	1125.72	363.43	0	Fixed	None
Model VIII	83			3.2521	Fixed	Left
Model XI	83			3.9954	Fixed	Mirror
Model V	83			4.1143	Fixed	Right
Model III	83			6.6217	Free	None
Model XIV	83			7.9200	Fixed	Both
Model IX	83			11.5286	Free	Left
Model XII	83			11.6086	Free	Mirror
Model VI	83			11.6651	None	Left
Model XV	83			16.9001	Free	Both
Model I	83			79.4648	None	None
Model IV	83			83.5923	None	Right
Model X	83			83.6182	None	Mirror
Model VII	83			83.6683	Free	Right
Model XIII	83			87.5992	None	Both
*Blue Frontal Streak Frequencies*						
Model I	109	819.78	242.94	0	None	None
Model IV	109			4.2714	None	Right
Model X	109			4.2715	None	Mirror
Model VII	109			4.2716	None	Left
Model III	109			4.3896	Free	None
Model XIII	109			8.7104	None	Both
Model IX	109			8.8155	Free	Left
Model XII	109			8.8160	Free	Mirror
Model VI	109			8.8540	Free	Right
Model XV	109			13.4679	Free	Both

**TABLE A9:** Species distribution model predictor variables for Steller's Jay morphotypes (Coastal, Interior, Rocky Mountain) based on climate and climate plus landcover.

Predictors	Climate only			Climate + landcover		
	Coastal	Interior	Rocky Mtn.	Coastal	Interior	Rocky Mtn.
Mean diurnal temperate range (BIO2)	✓	✓	✓	✓	✓	✓
Isothermality (BIO3)				✓	✓	✓
Temperature annual range (BIO7)	✓	✓	✓	✓	✓	✓
Precipitation of driest month (BIO14)	✓	✓	✓		✓	
Precipitation seasonality (BIO15)	✓	✓	✓	✓	✓	✓
Precipitation of warmest quarter (BIO18)	✓	✓	✓	✓	✓	✓
Precipitation of coldest quarter (BIO19)	✓		✓	✓		✓
Climatic Moisture Index (CMI)^a^			✓			✓
Emberger's pluviometric quotient^b^			✓			✓
Minimum temperature of warmest month	✓	✓	✓	✓	✓	✓
Mean monthly PET of driest quarter^c^	✓	✓	✓	✓	✓	
Mean monthly PET of wettest quarter^c^	✓		✓	✓		✓
PET seasonality^c^	✓	✓	✓	✓	✓	✓
Evergreen/deciduous needleleaf trees					✓	
Evergreen broadleaf trees						
Deciduous broadleaf trees						
Mixed/other trees						
Shrubs				✓	✓	✓
Herbaceous vegetation						✓
Regularly flooded vegetation						
Snow/ice						
Barren						✓

*Note*: Predictors were obtained from 33 bioclimatic variables (*biovars* function in the R package dismo v1.1‐4, Hijmans et al., 2017; R package envirem v2.1, Title & Bemmels, 2018) and nine landcover types (Tuanmu & Jetz, 2014).

^a^ Climatic Moisture Index is a metric of relative wetness and aridity.

^b^ Emberger's pluviometric quotient (embergerQ) is a metric to differentiate among Mediterranean climate types.

^c^ PET is a metric for potential evapotranspiration.

**TABLE A10:** Feature classes and smoothing parameters^a^ for MaxEnt, optimized via the corrected Akaike Information Criteria (AICc) model selection, by morphotype and variable type.

Morphotype	Climate only		Climate + landcover	
	Feature classes	Beta multiplier	Feature classes	Beta multiplier
Coastal	LQH	1	LQH	1
Interior	LH	1	LH	1
Rocky Mtn.	H	1	LH	1

^a^ Feature classes are different transformations of the environmental predictors (L, linear; Q, quadratic; H, hinge). The beta multiplier is a smoothing parameter that controls the level of overfitting of the model. Data show the combinations of feature class and beta multiplier that had the best AICc. See Warren et al. (2014) for further explanation of methodology.
